# Stable tendencies in spontaneous working memory usage during flexible behaviour

**DOI:** 10.3758/s13423-026-02955-y

**Published:** 2026-08-24

**Authors:** Levi Kumle, Amy X. Li, Joel Kovoor, Amelia G. Rock, Emily Hesselink, Ellen Jones, Méadhbh B. Brosnan, Anna C. Nobre, Dejan Draschkow

**Affiliations:** 1 Department of Experimental Psychology, University of Oxford, Oxford, UK; 2 School of Psychology, University College Dublin, Dublin, Ireland; 3 Wu Tsai Institute and Department of Psychology, Yale University, New Haven, CT USA; 4 Oxford Centre for Human Brain Activity, Wellcome Centre for Integrative Neuroimaging, Department of Psychiatry, University of Oxford, Oxford, UK

**Keywords:** Working memory, WM usage, Sensorimnemonic decisions, Individual differences, Naturalistic behaviour, Virtual reality

## Abstract

Unlike standard working memory (WM) tasks, real-world settings allow individuals to spontaneously adapt how much information to use from WM before resampling the environment. Here, we considered whether individuals exhibit distinct, trait-like tendencies in their spontaneous use of WM. Across three studies (*N* = 220) using an Object Copying Task, we show that some participants consistently exhibited greater WM usage than others. These tendencies were reliable, stable across time, and predicted individuals’ task efficiency and responses to changing task demands. Finally, spontaneous WM usage could only partially be explained by established measures of WM capacity and attentional control – two plausible predictors of natural WM usage. In summary, we find stable tendencies in individuals’ spontaneous WM usage, despite the variable nature of self-structured natural behaviour. Our results position inter-individual variability as an important dimension in understanding natural, memory-guided behaviour, highlighting the need to investigate its broader determinants and behavioural consequences.

**Supplementary Information:**

The online version contains supplementary material available at https://doi.org/10.3758/s13423-026-02955-y.

## Introduction

In everyday life, behaviour unfolds spontaneously, allowing for considerable flexibility in how we engage cognitive functions and structure actions to achieve goals. Consider, for example, building a self-assembly wardrobe: You encode instructions from the manual into working memory (WM), then search for a part based on the encoded information. Before placing the part, you are faced with a decision: Do you look back at the manual first, or do you rely on previously encoded information? During spontaneous behaviour, such self-determined “sensorimnemonic decisions” (Kumle, Nobre, et al., 2025b) flexibly structure *when* to use information in WM to guide behaviour and *how much* information to use before encoding anew. Importantly, such flexibility introduces both intra-individual variability (i.e., the same individual might approach the same task differently on different occasions or contexts) and inter-individual variability (i.e., one individual might prefer memorising and executing multiple steps at once, while another prefers checking the manual after each step). This raises a key question: despite the behavioural flexibility inherent in such tasks, do people show characteristic, trait-like tendencies in how they use WM to guide behaviour (WM usage)? If so, what are the determinants and behavioural consequences of these tendencies?

The need to consider behavioural flexibility when studying WM was highlighted by Ballard et al. (1995), who demonstrated that estimates of self-determined WM usage are lower than standard WM tasks would predict. That is, when information remains available in the external environment, individuals tend to encode from the environment often instead of using their WM up to capacity. Subsequent work has demonstrated that individuals flexibly adjust their spontaneous WM usage in response to contextual factors, such as the effort or time required to sample sensory information (Blaser & Kaldy, 2025; Draschkow et al., 2021; Gray et al., 2006; Grinschgl et al., 2021; Hardiess et al., 2011; Kumle et al., 2024; Kvitelashvili & Kessler, 2024; Li et al., 2023; Liang et al., 2025; Sahakian et al., 2023, 2024; Somai et al., 2020), distractions in the task environment (Grinschgl et al., 2023; Kumle et al., 2024), or the reliability and stability of external information (Droll & Hayhoe, 2007; Hoogerbrugge et al., 2024; Liang et al., 2025; Xu et al., 2025). However, most of this research has focused on group-level comparisons, examining how average WM usage changes across conditions. In contrast, we know little about potential individual tendencies in WM usage (Böing et al., 2024; Meyerhoff et al., 2021), with open questions about their stability across contexts and broader implications for task completion.

In part, this gap may reflect challenges of investigating trait-like WM usage in naturalistic tasks. WM-usage tasks are, by design, flexible and self-structured. While this enhances ecological validity, the resulting flexibility can lead participants to behave inconsistently across similar contexts. Moreover, the methodological complexity of measuring and isolating elemental cognitive functions in unconstrained settings (i.e., which allow a high degree of behavioural freedom) (Cisek & Green, 2024; Maselli et al., 2023; Nastase et al., 2020) further challenges the development of robust individual-difference measures. Thus, before examining whether the tendency in WM usage is *stable within* and *varies across* individuals, a central aim of this work is to test whether individuals’ tendency for WM usage in naturalistic settings can be captured reliably, despite its inherent flexibility.

If reliable differences in WM-usage tendencies exist, this raises the question of why some individuals use more WM than others. Accordingly, we also examine potential determinants of WM-usage tendencies. Specifically, we ask whether an individual’s WM-usage tendency in naturalistic settings can be explained by established cognitive measures in controlled experimental tasks. We focus on two candidate predictors. First, we consider WM capacity (for a review, see Luck & Vogel, 2013), a well-established correlate of overall cognitive abilities (e.g., Conway et al., 2002; Engle et al., 1999; Unsworth et al., 2014) and a seemingly logical constraint on WM usage (also see Böing et al., 2025; Kvitelashvili & Kessler, 2024; Meyerhoff et al., 2021). Second, differences in attentional processes have been linked to individual differences in WM capacity (e.g., Awh et al., 2006; Draheim et al., 2021; Engle & Kane, 2004; Unsworth et al., 2021; Unsworth & Spillers, 2010; Vogel et al., 2005) and higher-order cognitive functioning in naturalistic contexts (e.g., Draheim et al., 2022; Mashburn et al., 2023; Redick et al., 2016). Thus, we further explore whether attentional control – the ability to direct attention in a goal-oriented manner and overcome competing demands from distracting information (Burgoyne & Engle, 2020; Engle, 2018) – may also play a role in determining spontaneous WM usage in complex, self-structured tasks.

**Fig. 1:**
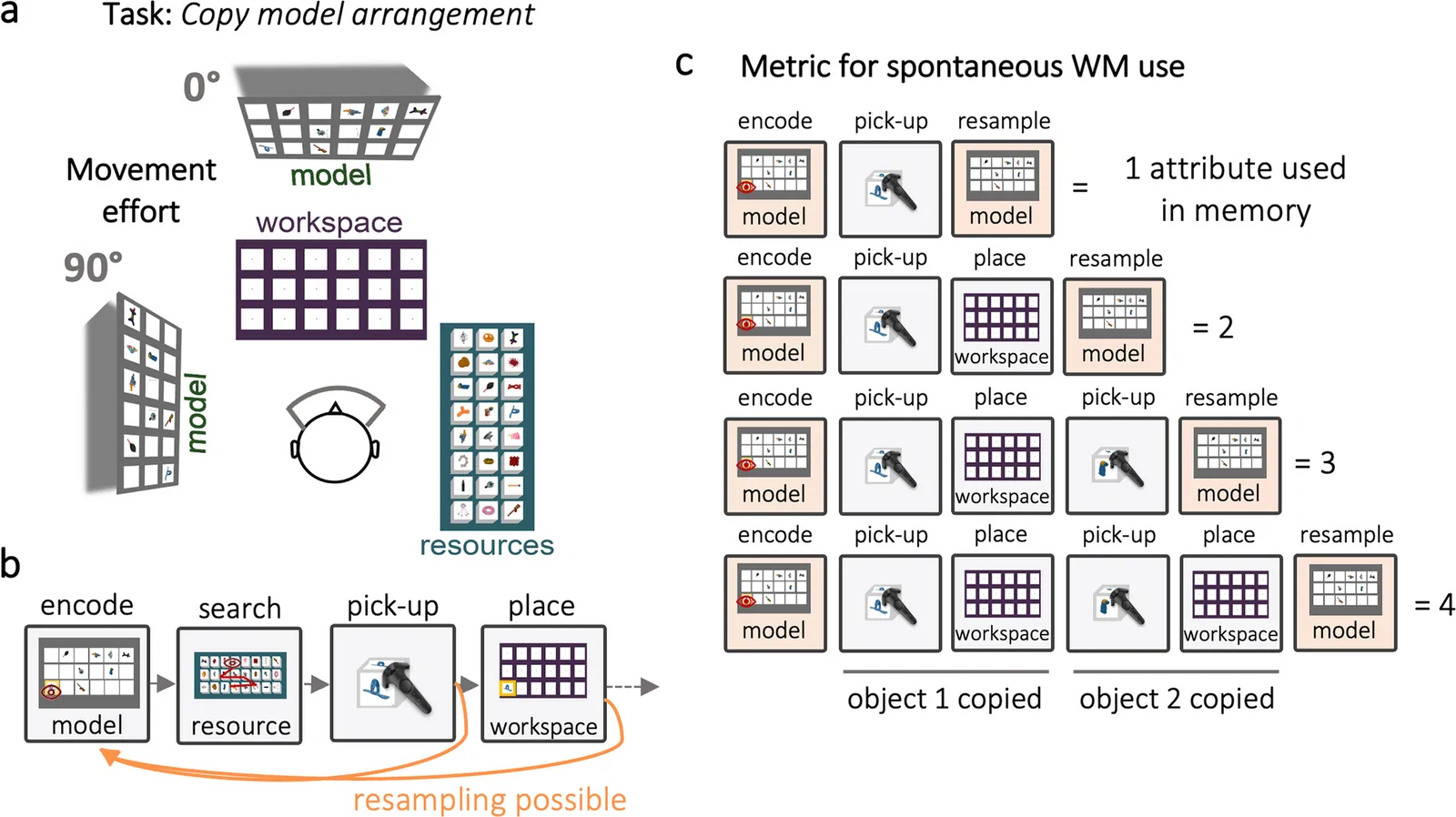
Overview of Object Copying Task and metric for working memory (WM) usage. **(a)** Participants were instructed to copy an arrangement shown in the model display by picking up objects from the resource table and placing them in the corresponding location on the workspace. The task included two movement-effort conditions (see Video 1). Specifically, the model was positioned either directly above the workspace (low movement effort) or rotated 90° (high movement effort). **(b)** Importantly, task-relevant sensory information remained accessible in the external environment, allowing participants to freely structure their behaviour. Specifically, participants could refer back to the model display at any point during the task (highlighted as “resampling possible” with orange arrows), whether after selecting an object from the resource pool or after placing it in the workspace. **(c)** An implicit metric for memory usage. Successfully copying an object requires using two representational attributes in memory: identity for pick-up and location for placement. Counting successful pick-ups (i.e., identity attribute used) and placements (i.e., location attribute used) between model viewings provided a metric for the number of attributes used in memory

To address these questions, we analysed data pooled from three separate studies (*N* = 220) in which participants completed the Object Copying Task in virtual reality (VR) (Draschkow et al., 2021; Kumle et al., 2024; Kumle, Kovoor, et al., 2025a). In our task, participants copied a model display by selecting objects from a resource pool and placing them into the corresponding location of the workspace (Fig. 1a, Video 1). That is, participants encoded task-relevant sensory information (i.e., target identities and locations) from the model display and could subsequently use this information in WM to guide the pick-up and placement of objects (Fig. 1b). Participants could structure their behaviour freely and refer back to the model display any time (Fig. 1b, “resampling possible”), thus allowing them to spontaneously choose how much information to use in WM to guide behaviour before sampling sensory information. Importantly, all three studies included a common experimental condition in which the model could be positioned at different locations within the virtual environment (0° or 90° relative to the workspace, see Fig. 1a). Manipulating the movement effort associated with sampling information from the model has been demonstrated to influence WM usage (Draschkow et al., 2021; Hardiess et al., 2011; Kumle et al., 2024), allowing us to compare WM usage in varying contexts. Further, a subset of participants (*N* = 30) completed the task at two different timepoints, allowing us to capture whether WM-usage tendencies remain stable over time. To link WM usage to attentional control and WM capacity, another subset of participants (*N* = 100) additionally completed established tasks indexing attentional control and WM capacity.

To foreshadow our findings, we demonstrate that individual tendencies in WM usage can be measured reliably, with some participants consistently using more WM than others. Importantly, these tendencies in WM usage extended to how individuals adapt to changing task demands and had implications for task performance. Finally, we show that WM-usage tendencies cannot simply be explained by traditional measures of WM capacity and attentional control. Overall, our results strongly position individual differences in WM usage as a distinct and informative dimension of naturalistic memory-guided behaviour.

## Method

The current article includes a combined analysis of three data sets: two published datasets (Kumle et al., 2024; Kumle, Kovoor, et al., 2025a) plus data from a separate, new preregistered study (https://osf.io/awj5y). All data sets stem from equivalent versions of an Object Copying Task in VR. In the following section, we first describe the shared characteristics between these three experimental studies. We then outline experimental details that were unique in the three studies, such as study-specific participant recruitment, procedures, and data preparation.

### Object-copying task

In all three studies, participants copied a model display by sequentially picking up eight objects from a resource pool and placing them into the corresponding location of the workspace (Fig. 1a**, **Video 1).

#### Virtual environment and procedure

Participants were equipped with a hand-held controller and a head-mounted display (HMD) before entering a virtual room containing the model display, resource pool, and workspace (see Fig. 1a and Video 1). Participants were informed that they had to copy the arrangement presented on the model display by picking up objects from the resource pool and placing them in the corresponding location of the workspace (Fig. 1b). No additional instructions were given beyond asking participants to solve the task as accurately as possible, allowing participants to freely structure their behaviour. Participants completed a minimum of four practice trials to get accustomed to the task and VR environment. Trials timed out after 45 s, and participants were informed about their remaining time through a backwards counting timer above the model.

The *model display* consisted of 18 white squares (arranged in a grid of three rows of six placeholders). Eight squares displayed target objects (i.e., to-be-copied objects). Target objects within the model were displayed in one of four rotations (90° increments) for increased variability, but the rotation of objects was not a relevant factor for correct placement. Participants always started the task facing the model. The *resource pool* contained 24 cubes (10 × 10 cm) arranged in three rows of eight cubes, including the eight targets in the model and an additional 16 distractors. The cubes and target slots in the model were overlaid with images of objects (different set of objects in each trial) from the Novel Object and Unusual Name Database (Horst & Hout, 2016), which are naturalistic while also unfamiliar and difficult to verbalize. The workspace consisted of an empty grid of 18 white placeholders in the same arrangement as the model (i.e., three rows of six).

Objects could be grabbed, moved, and placed by pulling, holding, and releasing the trigger button on the hand-held controller. Objects that were correctly placed “locked into” the workspace, with the placeholder’s outline turning green, indicating correct placement. To keep the number of objects in the resource pool constant, replacement objects (i.e., identical to the placed object) reappeared in the resource pool following correct placement. If an object was placed incorrectly, the placeholder would light up red, and the object would not lock into the workspace, prompting participants to correct their error. Participants were unable to pick up another object until the mistake was corrected. All other objects in the resource pool would disappear until the incorrectly placed object was correctly positioned or returned to the resource pool. This prevented errors from affecting subsequent task actions, as it forced participants to correct their mistakes before continuing.

To capture spontaneous WM usage in varying contexts, the model could be positioned at different locations within the virtual environment (0° or 90° relative to the workspace, Fig. 1a and Video 1). By varying the model location, we manipulated the movement effort associated with sampling information from the model (Draschkow et al., 2021). Reliance on memory in naturalistic tasks is typically low but increases when accessing sensory information becomes more effortful, making movement effort a useful tool to induce variability in WM usage and thus capture a broader range of naturalistic memory usage (Ballard et al., 1995; Draschkow et al., 2021; Gray et al., 2006; Hardiess et al., 2011; Kumle et al., 2024; Somai et al., 2020).

After a trial was completed (i.e., all eight target objects copied), the model, resource pool, and workspace disappeared, and participants entered a virtual breakroom. Once participants decided to start a new trial by pressing a button on the hand-held controller, the three task-relevant stations – model, resource pool, and workspace – re-appeared displaying the next trial arrangement.

#### Quantifying working memory (WM) usage

Spontaneous WM usage was quantified through a metric we refer to as *attributes used in WM* (see Fig. 1c) (Draschkow et al., 2021; Kumle et al., 2024; Kumle, Kovoor, et al., 2025a). Task behaviour within each trial was first segmented into behavioural sequences, each of which started with sampling information from the model (i.e., model-viewing period) and ended when the next model-viewing period started. The beginning of a model-viewing period was defined as the eye-tracking sample at which gaze first intersected with the model. A model-viewing period was considered concluded when gaze ceased intersecting with the model and did not return to it within 250 ms. To further qualify as a sequence, participants had to have looked at either the resource pool or the workspace in between gazing at the model. For instance, participants could gaze at the model, then turn to the resource pool and pick up an object, before returning to the model – which would mark the end of the current and beginning of the next sequence (Fig. 1c).

To track how much information participants used in WM in each behavioural sequence, we leveraged the fact that successfully copying one object within the Object Copying Task requires participants to use two representational attributes (the object’s *identity* and *location*). Specifically, searching for and picking up a target object requires using the object’s *identity*, and placing the object at the correct location requires using the object’s *location*. Attributes used in WM were therefore calculated by counting the correct pick-ups and placements of target objects within each behavioural sequence. For instance, participants might sample information from the model, pick up a target object, place the object, and then return to the model to gather additional sensory information (using two attributes in memory). Because item identity and location map onto distinct task-relevant actions, we treated them as separable, countable attributes, without making further assumptions about the relationship between identity-defining features and spatial information.

#### Quantifying overall task performance

Additionally, we quantified overall task performance through *display completion time.* Display completion time was calculated as the time elapsed between trial start and placement of all eight objects (i.e., until the moment of the eighth placement).

#### Data preparation

Here, we provide an overview for data preparation in all three data sets. For additional information on exclusions, see individual studies below.

For all analyses, we first excluded trials that were not completed before the time-out. For quantifying attributes used in WM, we excluded the first behavioural sequence in each trial. This exclusion was necessary since participants began each trial facing the model and needed to first orient themselves within the task environment. Additionally, sequences with an initial model viewing time (i.e., time between the start and end of each model-viewing period) of less than 50 ms or more than 3.5 standard deviations above an individual subject’s mean in each movement-effort condition were excluded. We then calculated the average number of attributes used in WM in each trial before calculating the average number of attributes used in WM for each participant in each movement-effort condition. Finally, we calculated participants’ average completion time by averaging completion times across all trials in each movement-effort condition.

## Studies 1 and 2: Re-analysis of Kumle et al. (2024, 2025a)

Next, we provide additional details about the Object Copying Task for the data used from the published studies by Kumle, Vo, Nobre, and Draschkow (2024) and Kumle et al. (2025a).

### Samples and overview

**Study 1:** In the study reported by Kumle, Vo, Nobre, and Draschkow (2024), 30 participants (*M*_age_ = 24.5, range_age_ = 18–32 years; 22 women, eight men, zero other; 26 right-handed, four left-handed, all self-reported) completed the VR Object Copying Task at two separate timepoints (7–12 days apart, *M*_t1-t2_= 7.4). Data for re-analysis were retrieved from https://doi.org/10.17605/OSF.IO/ZE5P3.

In addition to the two movement-effort conditions (0° vs. 90°, see Fig. 1a), participants completed trials in two distraction conditions (high vs. low distraction). In the low-distraction condition, distractor objects in the resource pool were covered with a white overlay set to 20% transparency and therefore appeared less salient. In the high-distraction condition, distractor objects in the resource pool were displayed with the same opacity as the target objects. Participants completed 28 trials in each combination of conditions at each timepoint (see Kumle et al., 2024, for a detailed overview). Only trials from the high distraction condition were analysed, as the stimulus parameters were equivalent to those in the other analysed data sets. Additionally, 87 trials (2.6%) were not completed before the time-out and were excluded from the current analyses.

**Study 2:** The study by Kumle et al. (2025a) included four experiments (one main and three supplementary experiments) in which participants completed the VR Object Copying Task. For the present analysis, we included Supplementary Experiments 1–3, which used the identical general layout as the other studies included in the present analysis. Data were retrieved from https://doi.org/10.17605/OSF.IO/6SWC8 and combined for all three experiments (*N* = 90, *M*_*age*_* =* 22.02*,* range_age_ = 18–39 years; 55 women, 34 men, one other/prefer not to say; 87 right-handed, three left-handed, all self-reported).

In addition to the two movement-effort conditions (0° vs. 90°, see Fig. 1a), half the trials included regularities (i.e., elements of the task environment such as the arrangement of target objects in the model repeated across trials). These repeating trials were interleaved with novel, non-repeating trials. Overall, participants completed 24 trials in each combination of movement effort and trial repetition (novel vs. repeated) condition. Only non-repeating trials were included for analysis, as these match trials in the other studies included in the present analysis. Additionally, 189 trials (4.6%) that were not completed before the time-out were excluded.

### Apparatus and virtual environment

In both Study 1 and Study 2, participants were equipped with an HTC Vive Tobii Pro VR system with a built-in binocular eye tracker and one wireless HTC Vive Controller in their dominant hand. The head-mounted display (HMD) featured dual OLED screens with a resolution of 1,080 × 1,200 pixels each, with a refresh rate of 90 Hz and a horizontal field of view of 100°, along with a vertical field of view of 3 × 110°. Gaze tracking occurred at a sampling rate of 90 Hz (synchronized with the HMD refresh rate) with an accuracy of approximately 0.5° visual angle. Participants performed a 5-point calibration after every 12–14 trials, or whenever they had removed the headset. Location in space for both the HMD and hand-held controller was tracked at a sampling rate of 90 Hz (synchronized with the HMD refresh rate) using two Lighthouse base stations emitting infrared pulses (60/s) detected by the sensors in the devices (37 infrared sensors in the HMD and 24 in the controller).

The virtual environment and experiment were programmed and run in Unity (version 2019.3; Unity Technologies) using the SteamVR Unity plugin (version 1.2.10; Valve Corporation).

## Study 3: Relating WM usage to WM capacity and attentional control

Data from the third study have not been previously published. Within this preregistered study (see https://osf.io/awj5y), we aimed to (1) confirm participant-specific patterns of spontaneous WM usage in an independent data set, and (2) investigate how measures of WM capacity and attentional control relate to spontaneous WM usage.

### Sample

We recruited 100 participants (*M*_age_ = 24.17, range_age_ = 18–51 years; 61 women, 36 men, three non-binary, 0 other/prefer not to say; 89 right-handed, 11 left-handed, all self-reported). All participants had normal (including colour vision) or corrected-to-normal vision (contact lenses). Participants provided informed consent before participation and received £15/h as compensation. The research protocol was approved by the Central University Research Ethics Committee, University of Oxford (#R64089/RE001). Six participants did not complete the study protocol (due to motion sickness or calibration issues during the VR task, non-attendance on the second testing visit) and were replaced.

Sample size planning was guided by an a priori power analysis and available resources. We conducted a power analysis for a structural equation modelling (SEM) approach using the pwrSEM shinyApp (https://yilinandrewang.shinyapps.io/pwrSEM/) and accompanying tutorial (Wang & Rhemtulla, 2021). Specifically, sample size planning was focused on detecting a significant regression coefficient between WM capacity or attentional control (estimated using a latent-variable approach described below) and the observed variable attributes used in memory (using respective models).

To derive a latent variable of WM capacity, factor loadings between the three WM-capacity tasks on the latent factor WM capacity were based on results presented in Draheim et al. (2021). Given the influence of factor loadings on statistical power (Wang & Rhemtulla, 2021), we decided to base our power calculations on slightly more conservative estimates (i.e., 10% smaller than reported in Draheim et al., 2021). For the model including a latent variable attentional control, factor loadings for the Squared attentional tasks and the latent factor attentional control were based on results in Burgoyne et al. (2023), which were overall comparable to the factor loadings reported in Draheim et al. (2021). That is, we decided to base sample size planning for both models on the same set of factor loadings (see shared materials for details). Residual variances were set automatically by the pwrSem shinyApp.

Given the available resources regarding collecting in-person VR data, we concluded that we would be able to test a maximum of 100 participants. The a priori power analysis confirmed that with this sample size and the above specified parameters, we would be adequately powered (power > 80%) to detect regression coefficients of > 0.35. We additionally confirmed that a sample size of 100 participants ensured adequate power for the planned regression analysis using G*Power (Faul et al., 2009). The selected sample size of *N* = 100 achieves power > 80% to detect standardized slopes of > 0.25.

### General procedure

Participants were invited to two in-person sessions (T1–T2 = 0–17 days, *M*_T1-T2_ = 4.3 days). Before the first testing session, participants provided informed consent and completed the long form of the *International Physical Activity Questionnaire* (IPAQ; Craig et al., 2003) remotely through Qualtrics (Qualtrics, Provo, UT, USA).

During the first testing session, participants completed the Object Copying Task in VR. Afterwards, participants reported their perceived physical and mental exertion during various stages of the VR task and filled out the *Simulator Sickness Questionnaire* (SSQ; Kennedy et al. (1993). In total, the first testing session lasted 90–120 min.

During the second testing session, participants completed a set of computer-based tasks in a group testing setting (max. eight participants). To assess WM capacity, participants completed the standard versions of the Operation Span, Rotation Span and Symmetry Span task (Unsworth et al., 2005). Each task was administered separately and lasted around 10–15 min. Participants additionally completed a set of attentional control tasks (i.e., the “Squared” Stroop, Simon, and Flanker tasks) (Burgoyne et al., 2023), which were always administered together and lasted around 10 min in total. The order of each WM task and the attention control task set was counterbalanced according to a balanced Latin square. Following the completion of all computer-based tasks, participants again reported their perceived physical and mental exertion throughout the testing session.

### Tasks and measures

#### VR object copying task

Participants completed eight rounds of ten trials (i.e., 80 trials in total). Each round consisted of two sub-blocks (five trials each), one in each movement-effort condition. The order of sub-blocks within rounds was randomised. Eighty trial configurations (i.e., arrangement for model display and resource pool) were pseudo-randomly generated (i.e., ensuring no duplicates). The assignment of configurations to movement-effort condition was counterbalanced across participants. The order of configurations within each movement-effort condition was randomized within each participant. A 5-point calibration was performed after each round or whenever participants took off the headset. Participants were able to take breaks after each sub-block. A mandatory break (5–10 min) was included after the fourth round, during which participants took off the HMD and could rest.

##### **Apparatus and virtual environment**

Participants were equipped with a Varjo VR-3 HMD, a wireless HTC Vive Controller in their dominant hand, and Vive body trackers on their waist and both ankles. The HMD included a display system with a uOLED focus area (70 Pixels Per Degree resolution and 1920 x 1920 pixels per eye) alongside an LCD peripheral area (30 Pixels Per Degree and a resolution of 2,880 × 2,720 pixels per eye). The overall horizontal field of view was 115°, with a display refresh rate of 90 Hz. Gaze data were captured using the built-in binocular eye tracker of the HMD at a sampling rate of 200 Hz, with three-dimensional (3D) gaze position determined by intersecting the gaze vector with objects in the virtual environment. Both the HMD and the controller locations were tracked at a sampling rate of 90 Hz, synchronized with the HMD refresh rate, using two Lighthouse base stations that emitted 60 infrared pulses per second, detected by 37 infrared sensors in the HMD and 24 in the controller.

The virtual environment and experiment were programmed and run in Unity (version 2022.3; Unity Technologies) using OpenXR (version 1.8.2; Khronos Group) and the Varjo Unity plugin (version 3.5.0; Varjo Technologies Oy) on a computer operated with Windows 10.

#### WM-capacity tasks

**Fig. 2:**
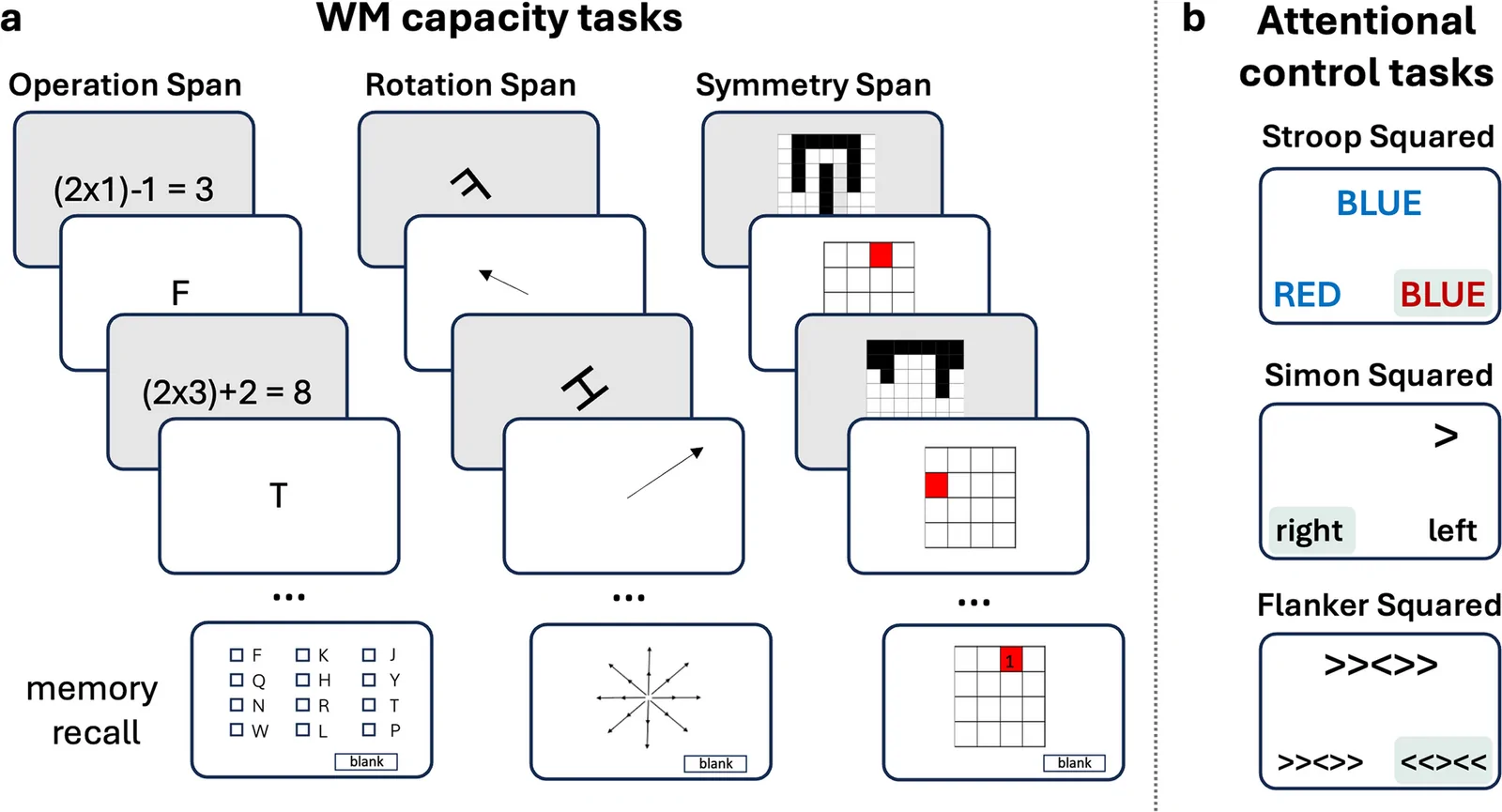
Working memory (WM)-capacity and attentional control tasks. **(a)** WM-capacity tasks introduced by Unsworth et al. (2005), consisting of alternating memory storage (white) and simple processing subtasks (grey). Participants had to remember and later report a series of letters (Operation Span), arrows (Rotation Span), or locations (Symmetry Span). Figure adapted from Fig. 1 in Draheim et al. (2018). **(b)** Attentional control tasks introduced by Burgoyne et al. (2023). In all three tasks, participants were shown a target at the top of the screen and two response options below. For the Stroop Squared task, the correct response was determined by the target’s colour and the semantic meaning of the response option. For the Simon Squared task, the correct response was determined by the target arrow’s direction and the meaning of the response option. For the Flanker Squared task, the correct response was determined by the direction of the target’s flanking arrows and the direction of the response option’s central arrow. Correct responses to the examples depicted in this figure are highlighted in green. Figure adapted from Fig. 7 in Burgoyne et al. (2023)

Working memory capacity was assessed using three automated complex span tasks (Fig. 2a), which consist of alternating memory storage and simple processing subtasks (Unsworth et al., 2005). The tasks were retrieved from https://englelab.gatech.edu/standardtasks.html and administered in the standard version.

##### **Operation span**

Participants had to remember a series of presented letters. During the processing subtasks, participants had to solve simple maths equations, deciding if the displayed solution was correct (e.g., (2 × 1) – 1 = 3). The presentation of letters and maths equations alternated until a variable set size of three to seven letters was achieved. Then, participants were asked to recall the letters by selecting the correct letters in their presented order out of 12 displayed letter options.

##### **Rotation span**

Participants had to remember a series of directional arrows in varying sizes. Participants were presented with one arrow at a time and had to memorise both its length and direction. During the processing subtasks, participants had to perform mental rotations, indicating whether a rotated letter was mirror-reversed. The presentation of arrows and rotated letters alternated until a variable set size of two to five was achieved. Afterwards, participants had to recall the arrows in the presented order. For memory recall, all possible angles were displayed, and participants had to click on the presented arrows in the correct order.

##### **Symmetry span**

Participants were tasked to remember a series of locations within a 4 × 4 matrix. To-be-remembered locations were highlighted in red. During the processing subtask, participants were presented with a 16 × 16 matrix of black and white squares and were required to determine whether the pattern exhibited vertical symmetry. The presentation of highlighted locations and symmetry judgements alternated until a variable set size of two to five locations was achieved. Then, participants had to recall the locations by clicking on the locations within an empty 4 × 4 matrix in the order they were presented.

For all three tasks, participants first familiarised themselves with both the memory and the processing subtasks. Specifically, participants completed a practice block for both subtasks separately in addition to a joined practice block. During test, set sizes were presented in a random order, ensuring that no set size was repeated until all sizes had been shown. Each set size was presented three times. The maximal response time for each processing subtask was set to the participant’s mean response time (as determined during the practice block of the processing subtasks) plus 2.5 standard deviations. For all processing subtasks, participants gave a binary true/false response. Participants were instructed to achieve a minimum overall accuracy of 85% on the processing subtasks, and their mean accuracy was displayed to them in the upper left corner of the screen throughout the task. For all memory storage parts, the dependent variable was the edit distance score, based on the number of changes required to edit the recalled sequence into the target sequence (Gonthier, 2023). The edit distance score was chosen over absolute or partial-credit scores (e.g., Unsworth et al., 2005) for its improved psychometric properties and increased test information (i.e., higher precision in discriminating participants’ abilities). For a detailed discussion of this scoring method and its advantages, see Gonthier (2023).

#### Attentional control tasks

To index attentional control, participants completed the *Three Squared Tests of Attentional Control* (Burgoyne et al., 2023), which were selected due to their demonstrated reliability and efficiency. For a comprehensive discussion and validation of these tasks, refer to Burgoyne et al. (2023). The tasks were retrieved from https://englelab.gatech.edu/squaredtasks.html, where the authors made them available.

##### **Stroop squared**

Participants were shown a target at the top of the screen and two response options below (targets and responses were always “RED” or “BLUE” in either red or blue colour; see Fig. 2b). As in the classic Stroop task, participants were tasked with attending to the colour of the target and not the semantic meaning of the word. However, participants had to select the response option based on the semantic meaning of the response option. That is, the correct response was determined by the colour of the target and the meaning of the response option.

##### **Simon squared**

Participants were shown a target arrowhead (either < or >; see Fig. 2b) appearing in the top left or right corner of the screen with equal probability, and two response options (either the word “LEFT” or the word “RIGHT) below. Participants were instructed to select the response option that states the direction toward which the arrow is pointing. That is, the correct response was determined by the direction of the target arrow and the meaning of the response option.

##### **Flanker squared**

Participants were shown a flanker array at the top of the screen with two response options below. All flanker arrays consisted of one central arrow flanked by two arrows on each side. Central and flanking arrows could point in the same (e.g., >>>>) or different (e.g., >><>>) direction (see Fig. 2b). Participants were instructed to select the response option in which the central arrow matched the flanking arrows of the target. That is, the correct response was determined by the directions of the flanking arrows of the target and the central arrow of the response option.

All three tasks consisted of four types of trials (intermixed throughout the task), based on the congruency between the target stimulus and response options. That is, trials could be fully congruent (i.e., both the target and response options were congruent, e.g., “RED” in red colour in the Stroop Squared task) or fully incongruent (e.g., “RED” in blue colour in both the target and response option). Additionally, the target could be congruent but the response options incongruent (“RED” in red colour for the target option but blue colour in the response option), or vice versa.

For each task, participants first completed a 30-s practice session, followed by a 90-s test session. Trials during the practice and test session were self-paced, and the next trial started immediately after participants responded to the current trial (i.e., the number of completed trials depended on participants’ reaction times). Participants received feedback after each trial. They earned a point for each correct response and lost a point for each mistake. The current score was displayed in the top right corner of the screen, while the remaining time was shown in the top left corner throughout the task. Performance was measured as the number of correct responses minus the number of incorrect responses.

##### **Additional questionnaires**

Given the physical demands of the VR Object Copying Task (e.g., prolonged standing and movement, including repeated head rotations), participants additionally completed a set of questionnaires probing their overall physical activity and exertion during the tasks. Specifically, participants completed the long form of the IPAQ (Craig et al., 2003). Participants indicated the time they have spent being physically active, separated into the domains of work-related, transportation, house/gardening, and leisure-time activity. On this basis, we calculated a continuous MET min/week following the IPAQ scoring protocol. MET minutes represent the amount of energy expended during physical activity. A MET represents a multiple of the estimated resting energy expenditure. Additionally, participants were asked about their perceived mental and physical exertion after both in-person testing sessions (for full details see *shared materials*). As an index of perceived exertion, we used participants’ responses to the items “*Please rate the level of mental (/physical) exertion you felt overall, where 1 is least exertion and 10 is most exertion.”*

Given that participants occasionally report discomfort (e.g., nausea, eye strain, or headaches) related to being exposed to virtual environments and the VR headset for extended periods (also see, e.g., Caserman et al., 2021; Yildirim, 2020), participants completed the *Simulator Sickness Scale* (SSQ; Kennedy et al., 1993) after the VR task to assess any impact on task performance. Within the SSQ, participants rate a range of symptoms (e.g., eye strain, dizziness, nausea) on a scale from 0 (none) to 3 (severe/strong symptoms). A total score was calculated based on the guidelines outlined in Kennedy et al. (1993).

### Data preparation

#### **VR object copying task**

We excluded 296 (3.7%) trials across participants that were not completed before the time-out.

#### **Attentional control and WM-capacity tasks**

Performance scores for all tasks were calculated using the *englelab* R-package (Tsukahara, 2022). Additionally, we calculated chance-level performance for all tasks. For the WM-capacity tasks, this was based on the processing – not storage – part of the task. Scores that were below chance were set to missing for all tasks (Stroop Squared: four scores excluded, no scores excluded in any other task). Next, we performed a two-pass outlier detection for scores above 3.5 standard deviations for each task. Detected outliers were set to missing. This criterion resulted in one score excluded in both the Flanker Squared and Operation Span tasks, and no scores excluded in any other task. Missing data could also occur when tasks crashed during testing (accounting for one missing score in the Rotation Span and Symmetry Span tasks). In addition to the exclusion criteria outlined in the preregistration, we also set scores to missing if participants indicated that they did not understand the instructions (Stroop Squared: one additional score excluded). Participants with missing data on more than one task for either attentional control or WM capacity would be removed from all further analysis, but the criterion did not apply to any participant. After data exclusion, we created composite scores for both attentional control and WM capacity by z-transforming scores for all attentional control and WM-capacity tasks and calculating the respective means.

#### **Additional questionnaires**

In line with the scoring guidelines of the IPAQ, MET min/week scores of participants who reported a daily activity time of over 16 hours were set to missing (two scores removed).

### Data analysis

All data were pre-processed and analysed in the R statistical programming language (version 4.2.2; R Core Team, 2022) using RStudio (version 2023.06.2; RStudio Team, 2023). Visualisations were done using the *ggplot2* package (Wickham, 2016).

#### Reliability of WM usage measures

##### Internal consistency

For each study, we calculated internal consistency estimates for average attributes used in WM in each movement-effort condition (and timepoint for Study 1) through split-half reliability corrected using the Spearman-Brown Prophecy formula. Specifically, trials within each condition (i.e., movement effort) and timepoint (for Study 1 only) were randomly split 1,000 times within participant, and split-half reliability was computed for each split using the Spearman–Brown correction, which were then averaged.

##### **Test-retest reliability**

Data from Study 1 were used to assess test-retest reliability. Test-retest reliability for each movement-effort condition was assessed using intraclass correlation coefficients (ICC) (Koo & Li, 2016) and the *psych* package (Revelle, 2022). ICC values < 0.5 indicate poor reliability, values between 0.5 and 0.75 indicate moderate reliability, values between 0.75 and 0.9 indicate good reliability, and values > 0.90 indicate excellent reliability (Koo & Li, 2016). First, we report reliability in terms of *absolute agreement* using a two-way, random-effects, single-measurement ICC (i.e., ICC 2,1 as specified in Koo & Li, 2016, and Shrout & Fleiss, 1979). However, because absolute agreement can be influenced by systematic biases (e.g., changes in memory usage between timepoints due to task-related learning), we primarily evaluate reliability in terms of *relative* consistency using a two-way mixed-effects measurement (i.e., ICC 3,1) (Liljequist et al., 2019; Shrout & Fleiss, 1979). Relative consistency reflects the degree to which the participants preserve their relative ranking.

#### Combined analysis: Participant-specific tendencies of WM usage

To increase statistical power and enhance the robustness of our findings, analyses on participant-specific patterns of WM usage were pooled across all three studies (*N* = 220). We additionally replicate the analyses within each independent data set (see Figs. S2–S4 in the Online Supplementary Material (OSM)).

To highlight intra-individual variability in spontaneous WM usage, we first replicated the effect of movement effort on spontaneous WM usage (Draschkow et al., 2021; Kumle et al., 2024). Specifically, we conducted a paired two-sample t-test between participants’ mean attributes used in memory in each movement-effort condition (i.e., 0° vs. 90°). Additionally, we calculated Cohen’s *d* and the corresponding 95% confidence interval as a measure of effect size using the *effectsize* package (Ben-Shachar et al., 2020)

To assess the consistency in the relative extent to which participants spontaneously used WM, we calculated Spearman's rank correlation between participants’ mean attributes used in WM in the 0° and 90° movement-effort conditions. This non-parametric analysis allowed us to evaluate the degree to which participants’ ranking of WM usage remains stable across the broader range of intra-individual variability in WM usage.

Further, we explored whether the extent to which participants adapt their WM usage to contextual factors differed depending on their relative level of WM usage. To this end, we related participants’ relative ranking in how much they spontaneously relied on WM to their context-dependent variability. To begin, we quantified context-dependent variability in WM usage by calculating the absolute difference in participants’ mean attributes used in WM between both movement-effort conditions (i.e., increase in WM usage when movement effort increased from 0° to 90°). Additionally, we quantified the proportional increase by dividing the absolute difference between conditions by participants’ mean attributes used in WM in the low movement-effort condition (0°). Using linear regression, we then predicted both absolute and proportional context-dependent variability by participants’ “baseline” WM usage in the 0° movement-effort condition. For data visualisation, we additionally categorised participants based on the relative extent to which they spontaneously relied on WM during the low movement-effort condition (i.e., 0°). Specifically, the mean values of attributes used in WM were divided into five equal groups based on the quintiles of the data, with each group representing a different level of spontaneous WM usage, ranging from low to high.

Finally, we related participants’ WM usage to their task performance as indexed by average completion time. Using linear regression, we predicted participants’ average completion time by their average attributes used in WM, separately for the 0° and 90° movement-effort conditions. For data visualisation, we again categorised participants based on their average attributes used in WM (separately for 0° and 90°) into five equal groups based on the quintiles of the data.

#### Study 3: Predicting WM usage through WM capacity and attentional control

Correlational research on individual differences, such as the preregistered analysis for Study 3, benefits from high variability across participants, as this aids in distinguishing among individuals (Hedge et al., 2018). As previous work (Draschkow et al., 2021; Kumle et al., 2024) found larger between-subject variance when movement effort was high compared to low, all analyses on the relationship between WM usage and WM capacity or attentional control were preregistered to focus on the 90°-movement-effort condition to increase sensitivity.

##### **Descriptive analysis**

To provide a descriptive overview of spontaneous WM usage in the Object Copying Task and each of the attentional-control and WM-capacity tasks, we calculated means, standard deviations, skew, and kurtosis of the respective outcome variables. To assess the reliability of the attentional-control and WM-capacity tasks, we calculated internal consistency using split-half reliability corrected with the Spearman–Brown prophecy formula. Specifically, we again averaged the split-half reliability over 1,000 random data splits. Further, we report Pearson’s correlations between all individual task scores and composite scores for attentional control and WM capacity.

##### **Regression analysis**

To assess the relationship between average spontaneous WM usage and attentional control and WM capacity, we performed linear regression analyses. In two separate models, the composite scores of attentional control and WM capacity were entered as continuous predictors of participants’ average attributes used in WM, respectively. To examine the unique contributions of attentional control and WM capacity in predicting participants’ average attributes used in WM, we additionally conducted a hierarchical regression analysis. Here, composite scores for attentional control and WM capacity were included as additive predictors into a model predicting participants’ average attributes used in WM. To determine whether the joint model significantly improved the explained variance compared to models including only attentional control or WM capacity individually, we performed a likelihood ratio (LR) test to compare the goodness of fit between the nested models.

##### Structural equation modelling

We additionally followed up the regression analysis with structural equation modelling to examine the relationships between attentional control and WM capacity on attributes used in WM on a latent level. All models were run using the *lavaan* R-package (Rosseel, 2012) using full information maximum likelihood estimation with robust standard errors. All variables were standardised (z-transformed) before entry into any structural equation model.

First, we tested whether all three attentional control tasks and WM-capacity tasks, respectively, load onto common latent factors (attentional control or WM capacity, respectively) using confirmatory factor analysis. The variance of the latent variables was fixed to allow for free estimation of each factor loading. We additionally tested a two-factor model with a latent factor for WM capacity and attentional control, where the two latent factors are allowed to co-vary. We then conducted two separate models that include a regression path from the latent factor of WM capacity or attentional control to the observed variable of participants’ average attributes used in WM. Finally, we conducted a combined model with latent factors for both WM capacity and attentional control, and regression coefficients from both latent factors to the observed variable of participants’ average attributes used in WM.

To assess model fit, we report χ2 as an index of absolute fit. Additionally, we report the comparative fit index (CFI) and Tucker–Lewis index (TLI), root mean square error of approximation (RMSEA) fit statistic, and the standardized root mean square residual (SRMR). For CFI and TLI, we interpret values >.90 or, ideally, >.95 as a good fit. For the RMSEA, we consider values smaller than.05 to be great and values less than.10 to be adequate. A SRMR with a value of less than.08 indicates good fit, following the guidelines of Hu and Bentler (1999).

##### **Exploratory analysis**

Potential relationships between spontaneous WM usage and overall physical activity, exertion during the tasks, and discomfort related to being exposed to virtual environments and the VR headset were examined by calculating Spearman’s rank correlation between participants’ mean attributes used in WM and MET min/week, SSQ, and exertion scores, respectively. In our preregistration, we outlined several potential follow-up analyses involving other aspects of the Object Copying Task (e.g., visual search during object pickup) that are not central to the current investigation and are instead part of future work. Correspondingly, these are not reported here.

## Results

We first present the combined analysis across all three datasets (*N* = 220), examining individual tendencies in WM usage, their reliability, and impact on task performance. Then, we report findings from the preregistered Study 3 (*N* = 100), where we assess whether WM-usage tendencies can be predicted by measures of attentional control and WM capacity.

### Measures of spontaneous WM usage are highly reliable and stable across time

Average attributes used in WM showed high internal consistency as a measure for participants’ tendencies in spontaneous WM usage (*r*_*sb*_ = 0.94–0.96 in both movement-effort conditions and all included studies; see Table S1 for Studies 1 and 2 and Table S2 for Study 3 (OSM)).

Further, analysis across days in Study 1 showed that participants’ tendencies in spontaneous WM usage were stable across time. That is, average attributes used in memory demonstrated good to excellent test-retest reliability in both movement-effort conditions (Fig. S1, OSM), both in terms of relative (0° movement effort: ICC_3,1_ = 0.76; 90° movement effort: ICC_3,1_ = 0.85) and absolute consistency (0° movement effort: ICC_2,1_ = 0.72; 90° movement effort: ICC_2,1_ = 0.79). Overall, these results suggest that individuals’ tendencies for spontaneous WM usage can be captured reliably and are stable across time, despite the self-structured nature and associated flexibility of the task.

### Stable tendencies in WM usage despite context-dependent inter-individual variability

In a combined analysis of all three studies (*N* = 220), we next explore whether participants differ in their spontaneous WM usage. For a replication of these analyses within each sub-study, see Figs. S2–S4 (OSM).

**Fig. 3:**
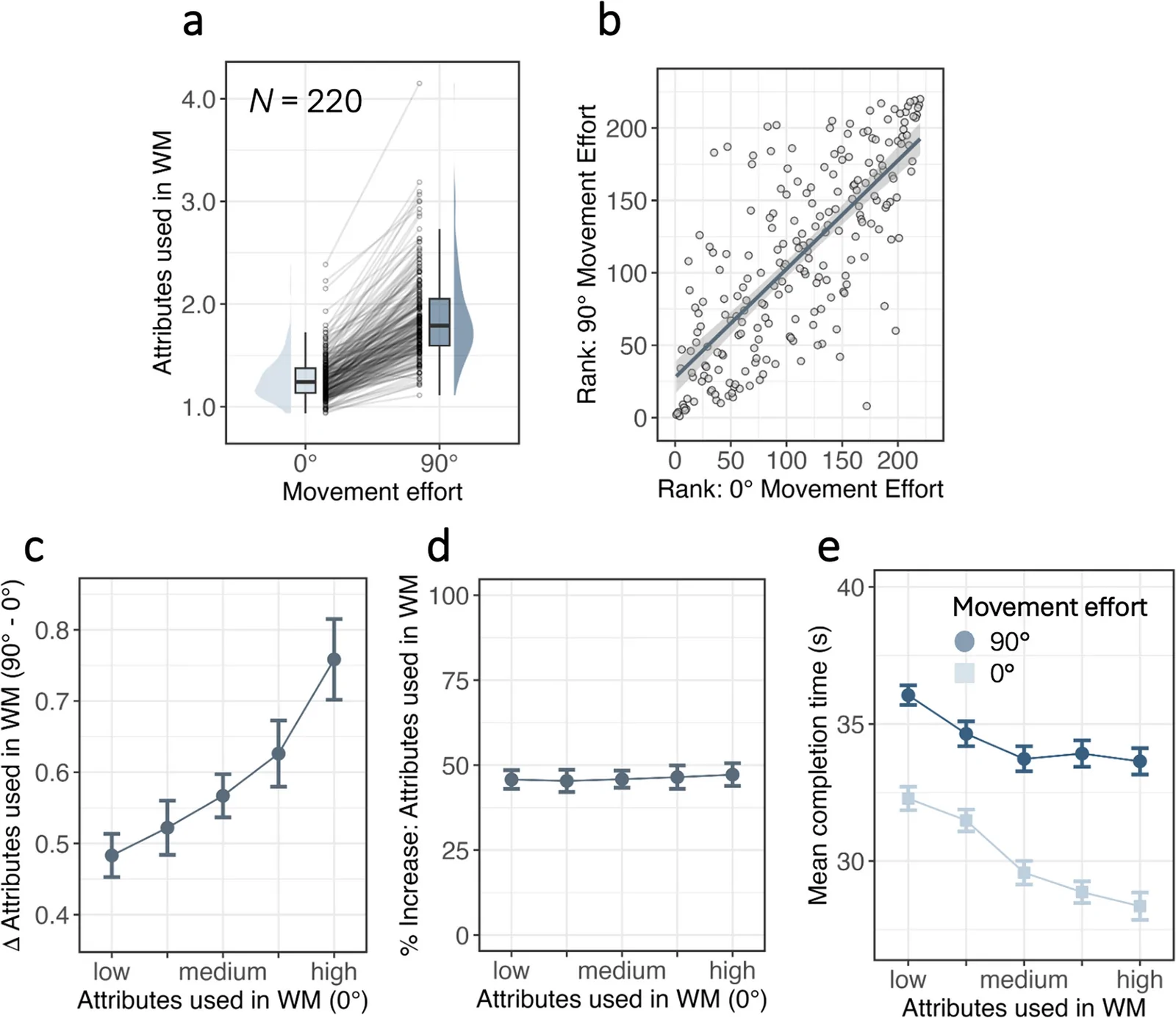
Combined analysis (*N* = 220): Characterising individual tendencies in spontaneous working memory (WM) usage. **(a)** Intra-individual variability in WM usage: Participants used more attributes in WM when movement effort was high (90°) compared to low (0°), replicating previous findings (Draschkow et al., 2021; Kumle et al., 2024). Dots and lines represent individual participant data. **(b)** Systematic differences in participants’ spontaneous WM usage: Some participants consistently used more WM than others in both movement conditions. Dots represent individual participant data. **(c)** Participants who tended to use more attributes in WM in low-movement trials had a stronger absolute increase in memory use when movement effort increased. Error bars depict standard errors (*N* = 44 per group). **(d)** No difference was observed in the relative increase in WM usage across participants with varying baseline WM usage tendencies. Error bars depict standard errors (*N* = 44 per group). **(e)** Consequence for task efficiency: Participants who used more attributes in WM on average were faster to complete the task in both movement conditions. Error bars depict standard errors (*N* = 44 per group)

Highlighting the intra-individual variability of spontaneous WM usage, we first replicate previous findings that WM usage adapts to contextual factors, such as the effort required to access task-relevant information in the external environment (Draschkow et al., 2021; Hardiess et al., 2011; Somai et al., 2020). Specifically, we compared participants’ WM usage when movement effort was high (90°) versus low (0°) (see Fig. 1a). Participants on average used more attributes in WM when movement effort was high (90°) compared to low (0°) (*t* = −30.31, *df* = 219, *p* < 0.001; *d* = −2.04, *CI*_95%_ = [−2.28, −1.81]; Fig. 3a).

Notably, we found systematic differences in participants’ tendencies of WM usage (see Fig. 3b). Specifically, participants’ relative WM usage (ranked average attributes used in WM) was highly correlated across movement-effort conditions (*Rho* = 0.73, *p* < 0.001; Fig. 3b). This indicates that some participants consistently used more WM than others, across a range of flexible WM usage.

### Differences in spontaneous WM usage predict adaptations to changing task demands

Finally, we explored potential relationships between participants’ relative WM usage and the extent to which they adjusted their WM usage in response to changing task demands (i.e., movement effort; see Figs. 3c and d). If these were unrelated, we would expect participants to adapt their WM usage to a similar extent across movement-effort conditions, regardless of their WM usage relative to other participants. To examine this, we quantified both the individual’s absolute and proportional change in WM usage between movement-effort conditions.

Participants with higher relative WM usage in low movement-effort trials showed a greater absolute increase in their WM usage in response to the increase in movement effort (ß = 0.53, *SE* = 0.08, *t* = 6.64, *p* < 0.001; Fig. 3c). However, this relationship disappeared when the increase in WM usage between movement-effort conditions was expressed proportionally relative to low-movement effort trials (ß = 4.59, *SE* = 6.39, *t* = 0.71, *p* = 0.47; Fig. 3d). Thus, participants with higher relative WM usage may not necessarily be more sensitive to contextual factors per se but still exhibit greater absolute changes in WM usage even though they began from higher baseline levels of spontaneous WM usage.

Overall, these larger absolute shifts in WM usage result in concrete differences in task behaviour between task demands, indicating that participants’ tendencies in spontaneous WM usage extended beyond how much information participants preferred to use in WM to include how they adapted their WM usage to changes in the environment.

### Consequences for broader task completion

Importantly, the degree of spontaneous WM usage introduces trade-offs that may impact task performance. Heavier reliance on information in WM reduces the frequency with which information needs to be sampled from the environment but may require additional time and effort for encoding (i.e., participants encode more information at once) (Draschkow et al., 2021; Kumle et al., 2024). Conversely, minimal use of WM may save time during encoding, but requires more frequent sampling from the environment. Therefore, we additionally sought to examine how individual differences in WM usage could influence overall task performance (i.e., the efficiency with which participants complete the task)

Participants who used more attributes in WM on average were faster to complete the task, both when movement effort was high (ß = −1.50, *SE* = 0.48, *t* =−3.10, *p* = 0.002) and when it was low (ß = −5.50, *SE* = 0.95, *t* = −5.80, *p* < 0.001), highlighting that participants’ tendencies of WM usage have consequences for task efficiency (see Fig. 3d).

### Study 3: Relating WM usage, attentional control, and WM capacity

Next, we turn to results from Study 3, assessing the potential relationships between tendencies in spontaneous WM usage and individual differences in WM capacity and attentional control. Descriptive statistics for all WM-capacity and attentional-control tasks can be found in Table S2 (OSM). Correlations between scores from all tasks – including the Object Copying Task and composite scores for WM-capacity and attentional control tasks – can be found in Table S3 (OSM). In brief, the attentional control tasks show excellent reliability (*r*_*sb*_* =*.93 ˗.95, see Table S2, OSM) and are correlated with each other (r =.52 ˗.59), replicating findings from Burgoyne et al. (2023). Reliability for the three WM-capacity tasks was moderate (*r*_*sb*_* =*.56 ˗.70), and all three tasks correlated with each other (r =.43 ˗.60).

**Fig. 4:**
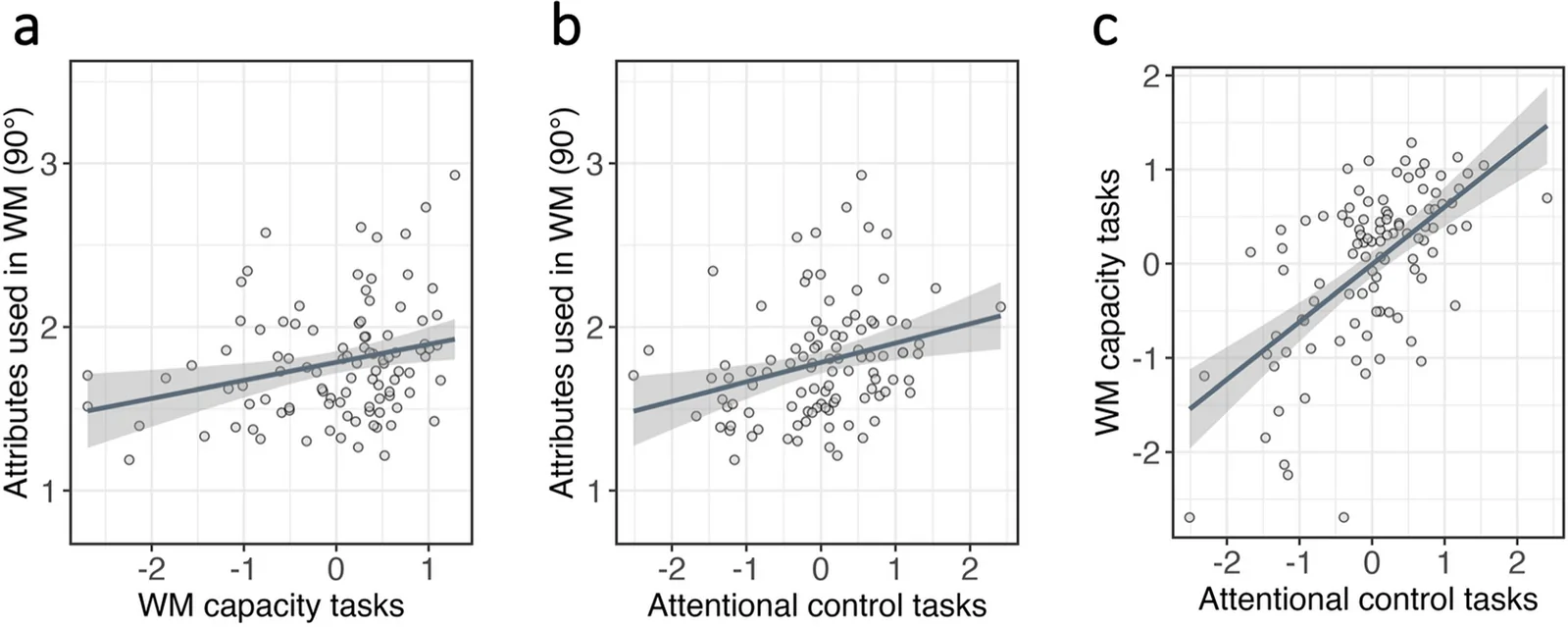
Relationships between working memory (WM) capacity, attentional control, and average attributes used in WM. **(a)** Composite scores for performance in the WM-capacity tasks predict average attributes used in WM: Participants who showed higher performance in the WM-capacity tasks used more attributes in WM. **(b)** Composite scores for performance in the attentional control tasks predict average attributes used in WM: Participants who showed higher performance in the attentional control tasks used more attributes in WM. **(c)** Composite scores for performance in the WM-capacity and attentional-control tasks were highly correlated, indicating that these tasks did not capture unique variances. Dots represent individual participant data (*N* = 100)

Composite scores for WM-capacity tasks predicted participants’ average attributes used in WM (*β* = 0.10, *SE* = 0.04, *t* = 2.56, *p* = 0.01; see Fig. 4a). Similarly, composite scores for attentional control tasks predicted participants’ average attributes used in WM (*β* = 0.11, *SE* = 0.04, *t* = 2.71, *p* < 0.01; see Fig. 4b). That is, participants who showed higher performance in the WM-capacity and attentional-control tasks tended to spontaneously use more attributes in WM during the Object Copying Task.

However, composite scores for performance in the attentional control and WM-capacity tasks were highly correlated (*r* =.61, *p* < 0.001; see Fig. 4c and Table S3, OSM), complicating the interpretation of the results from these tasks in terms of capturing unique variance. Relatedly, when both attentional control and WM capacity were entered as predictors in a joint model, neither attentional control (*β* = 0.07, *SE* = 0.05, *t* = 1.44, *p* = 0.15) nor WM capacity (*β* = 0.06, *SE* = 0.05, *t* = 1.16, *p* = 0.25) significantly predicted participants’ average attributes used in WM. Additionally, the joint model did not explain significantly more variance than the model with just attentional control (ΔR^2^ = 0.01, χ^2^(1) = 0.16, *p* = 0.24) or WM capacity (ΔR^2^ = 0.02, χ^2^(1) = 0.24, *p* = 0.15) as a predictor. Taken together, our results suggest that established measures of WM capacity and attentional control predict – albeit only partially – spontaneous WM usage. The strong correlation between attentional control and WM-capacity tasks precludes us from interpreting their distinct influences. We further replicated these findings using a structural equation modelling approach (see Figs. S5 and S6, OSM).

Above and beyond identifying separable contributions of the two measures, the model that included both attentional control and WM capacity as predictors only explained ~8% of variance. This suggests that spontaneous WM usage can only partially be explained by established measures of WM capacity and attentional control.

### Exploratory results

We found no significant relationships between the average of attributes used in WM and measures of physical activity, VR-induced discomfort, or exertion (MET min/week, SSQ, exertion scores; all *p* > 0.05, see Fig. S7, OSM). Thus, physical demands of the VR Object Copying Task are unlikely to account for differences in spontaneous WM usage.

## Discussion

Our findings show that reliable and stable, trait-like differences in WM usage emerge in highly flexible, self-structured behaviour. These findings highlight that understanding naturalistic memory-guided behaviour requires considering not only the underlying memory capacities and processes, but also how different individuals spontaneously use WM across contexts.

The high reliability and trait-like tendencies of WM usage may initially seem surprising. First, allowing participants to spontaneously self-structure an extended task – instead of following a controlled task sequence as in standard experimental tasks – might have led to state-dependent, inconsistent behaviour. Second, many experimental tasks that produce reliable group-level effects fail to capture reliable individual differences (for a meta-analysis on consistent group-level effects in the Object Copying Task, see Qing et al., 2025). This “reliability paradox” reflects the fact that group-level effects are strongest when between-participant variance is low, whereas reliable measurement of individual differences depends on substantial between-participant variability (Hedge et al., 2018).

Against this background, we argue that reliable and stable WM-usage tendencies emerged not *in spite of* the possibility to self-structure behaviour, but *because* of it. That is, allowing participants to organise their behaviour in a self-determined rather than task-determined manner enabled them to adopt individually preferred cognitive strategies, increasing between-participant variability and resulting in WM-usage tendencies that are reliable, stable, and appear trait-like. This interpretation aligns with other naturalistic tasks that offer varying degrees of self-structuring and strategic freedom, such as free-viewing and visual search. For instance, eye movements in such tasks have shown consistent, participant-specific patterns (Boot et al., 2009; Castelhano & Henderson, 2008; Henderson & Luke, 2014; Rayner et al., 2007). Here, we extend such stable cognitive tendencies during spontaneous behaviour to a higher-level cognitive process (see also Meyerhoff et al., 2021), further highlighting the need to consider cognitive functions in flexible, naturalistic settings that permit self-structured behaviour.

Importantly, we show that WM-usage tendencies go beyond *how much* information people tend to use in WM; they also predict task performance and adaptations to changing task demands. Differing WM-usage tendencies may have additional consequences. For instance, existing group-level studies suggest that higher WM usage increases subsequent incidental memory for information encountered during the task (Grinschgl et al., 2021; Morgan et al., 2009), with exploratory participant-level analyses pointing in the same direction (Grinschgl et al., 2021). Importantly, such immediate effects may accumulate over time, leading to broader, longer-term implications. For example, individuals who routinely choose to use more information in WM might benefit from retaining more information in contexts like education (e.g., while using instructions from a worksheet or textbook to complete a task). Exploring both immediate and long-term consequences of WM-usage tendencies will be important for gaining a comprehensive understanding of WM-usage profiles.

Further, it remains important to establish whether individual WM-usage tendencies generalise across tasks indexing naturalistic WM usage. While the Object Copying Task is arguably the most widely used task for exploring WM usage in naturalistic settings (Ballard et al., 1995; Draschkow et al., 2021; Grinschgl et al., 2021; Hardiess et al., 2011; Sahakian et al., 2023; Somai et al., 2020), it is only one of several tasks developed in response to growing interest in this area (Droll & Hayhoe, 2007; Kvitelashvili & Kessler, 2024; Li et al., 2023; Liang et al., 2025). Common group-level effects (e.g., effort/cost of resampling) have been demonstrated within different tasks designed to index naturalistic WM usage (e.g., see Qing et al., 2025, for a meta-analysis of group-level effects within the Object Copying Task), but little work has compared the consistency of individual WM-usage tendencies across tasks.

One relevant exception comparing behaviour across tasks is a study by Meyerhoff et al. (2021), which compared WM usage in the Object Copying Task with *intention offloading* (i.e., creating external reminders for delayed intentions; also see Gilbert, 2015a, 2015b, and Gilbert et al., 2020) in a prospective memory task. They found no correlation in behaviour across the two tasks (i.e., higher WM usage was not associated with more frequent reliance on memory for prospective actions). The finding taps into an important conceptual distinction. Intention offloading captures scenarios where memorising information can be avoided by actively “offloading” it into a manipulable external environment, effectively transferring information from memory into the environment. In contrast, WM usage is essential for task completion in the Object Copying Task; but individuals can flexibly coordinate *how much* memory information is used to guide behaviour at a time, before re-encoding from a stable and accessible environment. Here, the information in the external environment is not used to replace memory but to “onload” information *into* memory. Directly comparing individual behaviour across multiple WM-usage tasks – and distinguishing these from related constructs like cognitive/intention offloading – will be essential for strengthening the interpretation of WM-usage tendencies as conceptually distinct and trait-like.

To explore *why* some participants use more WM than others, we considered two promising predictors, WM capacity and attentional control. Our results suggested that WM usage can only partially be explained by established measures of WM capacity and attentional control, but the high correlation between these measures limited our ability to disentangle their unique contributions to WM-usage tendencies. This correlation is in line with prior work showing strong associations between WM capacity and attentional control (Awh et al., 2006; Engle, 2018; Unsworth et al., 2021; Vogel et al., 2005). Importantly, we indexed WM capacity using complex span tasks, which combine WM storage with ongoing processing of secondary information (Engle et al., 1999), instead of simpler storage-specific tasks. On one hand, this makes them more comparable with our WM usage task (i.e., Object Copying Task), in which participants also had to maintain task-relevant information while simultaneously engaging in ongoing secondary processing (e.g., searching for targets on the resource table among task-irrelevant distractor objects). On the other hand, the substantial attentional demands of complex span tasks likely contributed to the correlation observed between WM capacity and attentional control measures (e.g., Conway et al., 2005; Shipstead et al., 2015). It is therefore possible that different patterns of results may have emerged had we used more storage-specific WM measures with reduced attentional control demands.

Our findings further align with mixed results from previous work examining the link between WM usage and WM capacity (Böing et al., 2025; Grinschgl et al., 2021; Kvitelashvili & Kessler, 2024; Meyerhoff et al., 2021). The weak link between usage and capacity may reflect methodological factors, such as differences in the specific tasks used to index capacity or limited sensitivity to individual differences. However, we believe these findings also provide substantive reasons to propose that WM capacity and WM usage can be dissociated. Critically, our metric of WM usage does not capture how much information participants encoded or maintained, but rather how much of that information they used to guide behaviour before encoding anew. Prior work shows that these are not equivalent: participants may hold “usable” information in WM and yet choose to re-encode from the environment (Sahakian et al., 2023).

Overall, WM-usage tendencies are likely shaped by a multitude of factors. For example, motivational priorities and dispositional traits such as error sensitivity or perfectionism (e.g., Boksem et al., 2006; Stahl et al., 2015) may lead some to prioritise accuracy and therefore re-sample external information more readily rather than using information in WM. In contrast, others may prioritise speed (e.g., Draheim et al., 2016; Evans et al., 2017; Hedge et al., 2019) and adopt more WM-intensive strategies to minimise additional re-sampling (and the associated time cost). Importantly, apart from the 45-s time limit, participants received no instructions to prioritise either speed or accuracy in the WM-usage task, leaving room for individual preferences. Further, metacognitive factors such as beliefs about one’s WM abilities (e.g., confidence in memory abilities) have been shown to affect cognitive offloading behaviour (Gilbert, 2015b; Gilbert et al., 2020) and may similarly shape spontaneous WM usage. However, initial evidence by others points in the opposite direction: subjective memory efficacy did not predict spontaneous WM usage in a sample of older adults (Böing et al., 2025). Although this discrepancy requires additional investigation, it suggests that cognitive offloading and spontaneous WM usage may be mechanistically distinct. Additionally, demographic factors such as participants’ age may also influence WM-usage tendencies. For example, Touron and Hertzog (2004) found that older compared to younger adults were less likely to adopt memory-intensive task strategies. Finally, WM-usage tendencies could reflect consistent state factors such as alertness or fatigue, rather than enduring cognitive traits. Importantly, these factors could influence WM-usage tendencies through different mechanisms. Individuals may differ in how much information they initially encode and maintain, how much of maintained information they used, or both. We hope our findings lay the groundwork for systematically disentangling these in future research.

## Supplementary Information

Below is the link to the electronic supplementary material.

Supplementary file1 (PDF 2021 KB)

Supplementary file2 (MP4 18977 KB)

## Data Availability

Secondary data were retrieved from the Open Science Framework at https://doi.org/10.17605/OSF.IO/ZE5P3 (Study 1) and https://doi.org/10.17605/OSF.IO/6SWC8 (Study 2) and have been reshared with the authors’ permission (https://osf.io/wpf3s). Data and original materials for Study 3 are available at https://osf.io/wpf3s.

## Code availability

Code for all analysis is publicly available via the Open Science Framework at https://osf.io/wpf3s.

## Declarations

### Open practices statement

The data and materials for all experiments are available via the Open Science Framework (https://osf.io/wpf3s/) and Study 3 was preregistered (https://osf.io/awj5y).
