# Supplementary material for: Stable tendencies in spontaneous working memory usage during flexible behaviour
Source: Psychon Bull Rev. 2026 Aug 24;33(7):215. doi: 10.3758/s13423-026-02955-y (PMC13503484; doi:10.3758/s13423-026-02955-y)
Supplement: Supplementary file 1 [file 13423_2026_2955_MOESM1_ESM.pdf]

Table S1. Descriptive statistics for attributes used in WM in data set 1 and 2.

Dataset	Condition	N	Mean	SD	Reliability
Study 1	T1: 90° movement effort	30	2.02	0.47	0.95
	T2: 90° movement effort	30	2.20	0.47	0.95
	T1: 0° movement effort	30	1.31	0.24	0.96
	T2: 0° movement effort	30	1.39	0.26	0.96
Study 2	90° movement effort	90	1.86	0.46	0.96
	0° movement effort	90	1.22	0.20	0.94

Note. Reliability estimates represent mean of N = 1000 random split-half reliability corrected using Spearman-Brown prophecy formula.

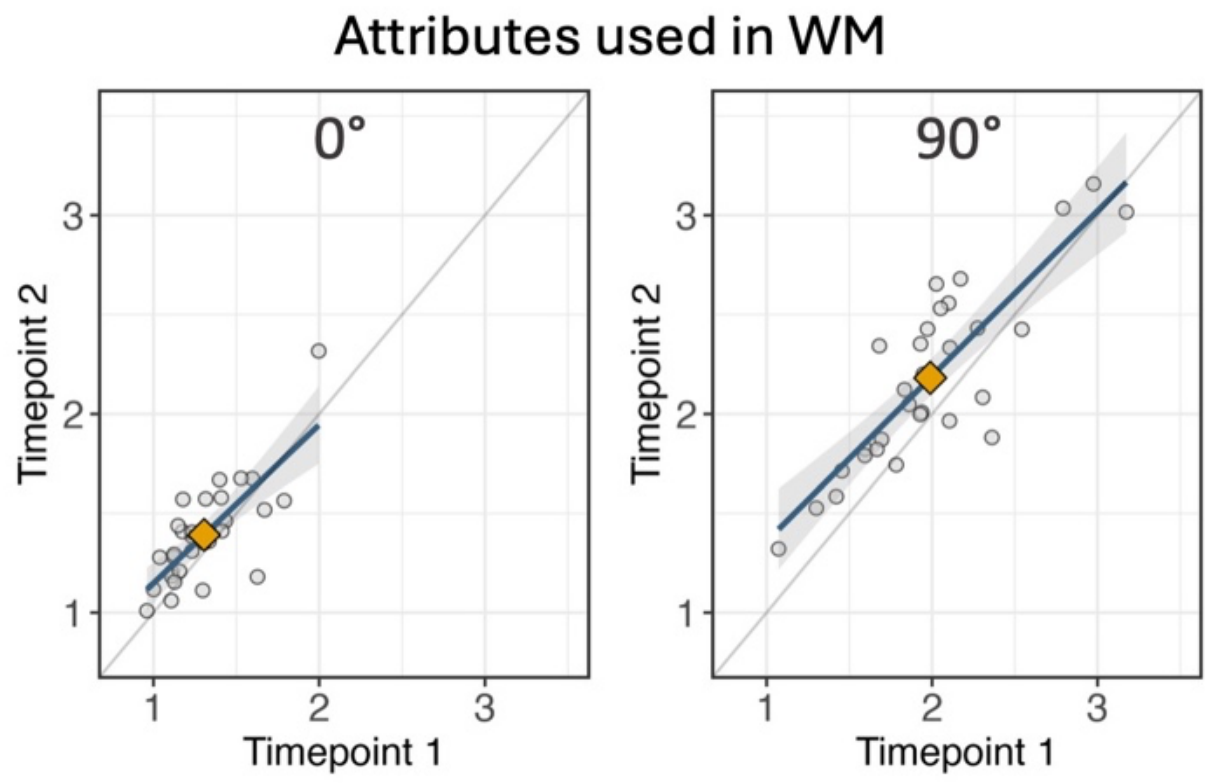

Figure S1. **Test-retest reliability.** In *S*tudy 1*(N = 30)*, participant completed the Object Copying Task twice ($M_{t1\text{-}t2}$= *7.4* days apart*)*. Participants' average attributes used in WM showed high test-retest reliability in both movement effort conditions*,* both in terms of relative (0° movement effort: ICC3,1 = 0.76; 90° movement effort: ICC3,1 = 0.85) and absolute consistency (0° movement effort: ICC2,1 = 0.72; 90° movement effort: ICC2,1 = 0.79). Dots represent individual participant data. Orange diamonds depict sample means.

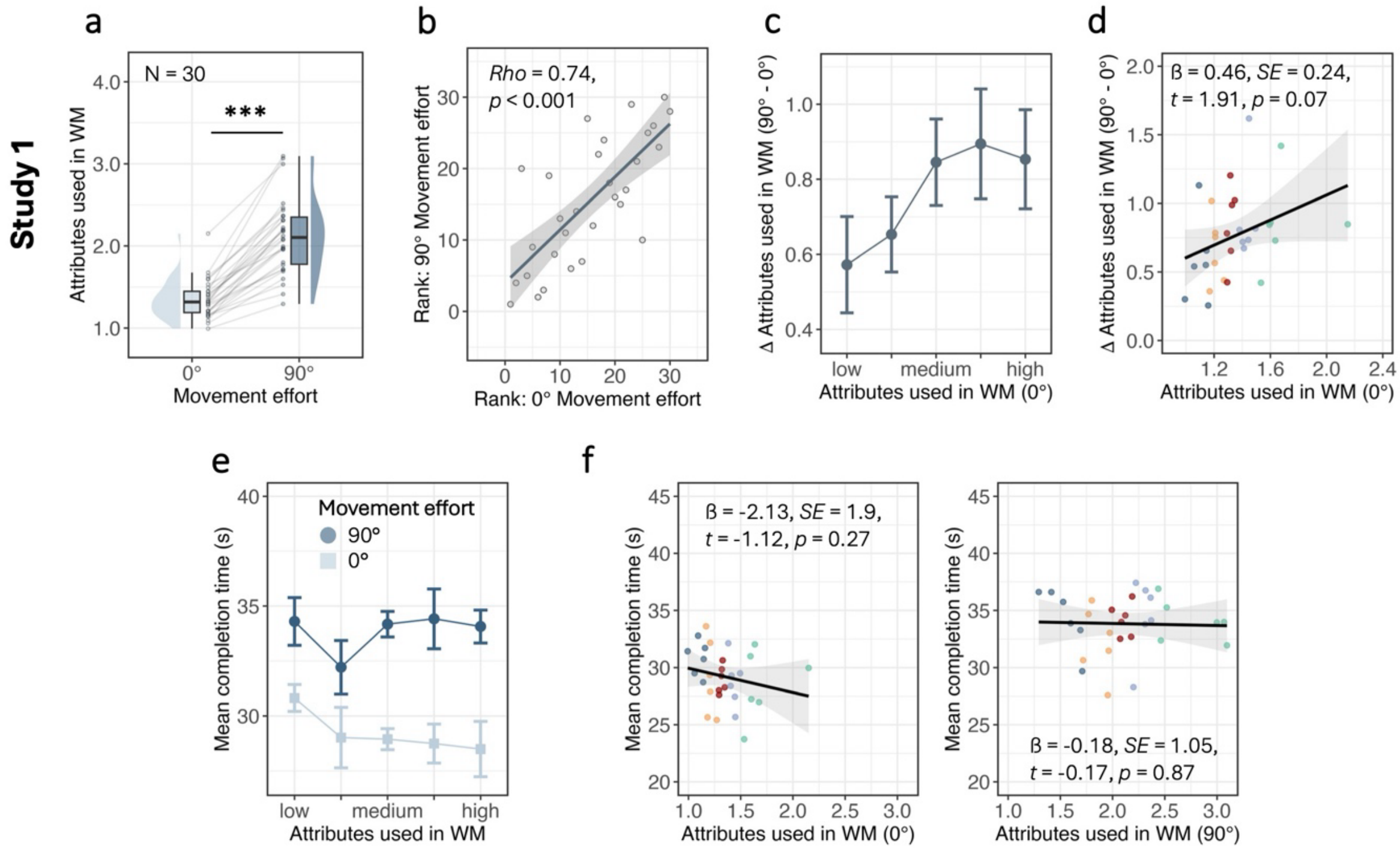

***Figure S2*. Participant specific tendencies in WM-usage, replication with data from Study 1 only. a)** Intra-individual variability in WM-usage: Consistent with the combined analysis, participants in Study 1 used more attributes in WM when movement effort was high (90) compared to high (t = -13.36, df = 29, p < 0.001; d = -2.44, CI95% = [-3.15, -1.71]). Dots and lines represent individual participant data. **b)** Systematic differences in participants spontaneous WM-usage: Consistent with the combined analysis, participants' relative WM-usage (ranked average attributes used in WM) was highly correlated across movement effort conditions (*Rho* = 0.74, p < 0.001) in Study 1. Dots represent individual participant data. **c) and d)** Unlike the combined analysis, we found no meaningful relation between participants' WM-usage in low-movement effort trials and their increase in WM-usage when movement effort increased (β = 0.46, *SE* = 0.24, t =1.91, p = 0.07). Panel c visualises participants' categorical WM-usage in low-movement effort trials. Here, error bars depict standard errors (N = 6 per group). Panel d depicts participants' WM-usage in low-movement effort trials without categorical grouping. Here, dots represent individual participant data. Colour indicates matching to categorical groups. **e) and f)** Unlike the combined analysis, we found no meaningful relationship between participants average attributes used in WM and their task efficiency as indexed by average trial completion time, both when movement effort was low (β = -2.13, *SE* = 1.9, t =-1.12, p = 0.27) and high (β = -0.18, *SE* = 1.05, t =-0.17, p = 0.87). Panel e visualises participants' WM-usage as categorical means (N = 6 per group). Here, error bars depict standard errors. Panel f depicts participants' WM-usage without categorical grouping. Here, dots represent individual participant data. Colour indicates matching to categorical groups.

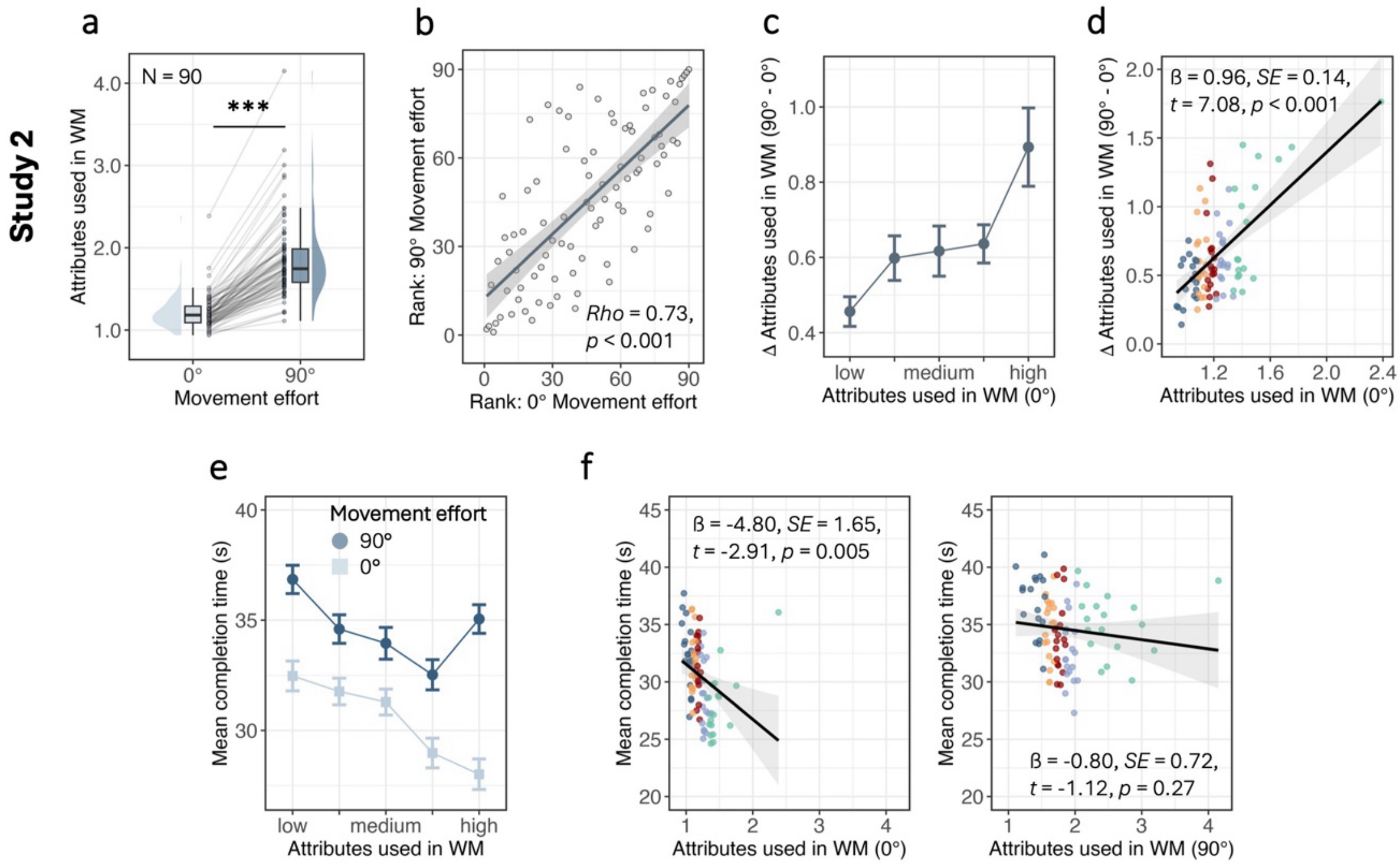

Figure S3. **Participant specific tendencies in WM-usage, replication with data from Study 2 only. a)** Intra-individual variability in WM-usage: Consistent with the combined analysis, participants in Study 2 used more attributes in WM when movement effort was high compared to low (t = -19.3, df = 89, p < 0.001; d = -2.03, CI95% = [-2.40, -1.67]). Dots and lines represent individual participant data. **b)** Systematic differences in participants spontaneous WM-usage: Consistent with the combined analysis, participants' relative WM-usage (ranked average attributes used in WM) was highly correlated across movement effort conditions (*Rho* = 0.73, p < 0.001) in Study 1. Dots represent individual participant data. **c) and d)** Consistent with the combined analysis, we found a positive relation between participants' WM-usage in low-movement effort trials and their increase in WM-usage when movement effort increased (β = 0.96, SE = 0.14, t =7.08, p < 0.001). Panel c visualises participants' categorical WM-usage in low-movement effort trials. Here, error bars depict standard errors (N = 18 per group). Panel d depicts participants' WM-usage in low-movement effort trials without categorical grouping. Here, dots represent individual participant data. Colour indicates matching to categorical groups. **e) and f)** Partially consistent with the combined analysis, we found a negative relationship between participants average attributes used in WM and their task efficiency as indexed by average trial completion time when movement effort was low (β = -4.80, SE = 1.65, t =-2.91, p = 0.005) but not when movement effort was high (β = -0.80, SE = 0.72, t =-1.12, p = 0.27). This, however, seems largely to be driven by a single outlier participant (~5 SD above condition mean in both movement effort conditions). Upon removing this participant, we find a negative relationship between participants average attributes used in WM and their task efficiency as indexed by average trial completion time when movement effort was low (β = -11.22, SE = 1.85, t =-6.06, p < 0.001) and high (β = -1.84, SE = 0.82, t =-2.23, p = 0.03). Additionally, as suggested by panel e and previous work (Draschkow et al., 2021), a higher order polynomial trend might contribute to the relationship between participants' average attributes used in WM and completion time. While this has not been the focus of the current investigation, it overall supports the link between participants spontaneous WM-usage and their task efficiency. Panel e visualises participants' WM-usage as categorical means (N = 18 per group). Here, error bars depict standard errors. Panel f depicts participants' WM-usage without categorical grouping. Here, dots represent individual participant data. Colour indicates matching to categorical groups.

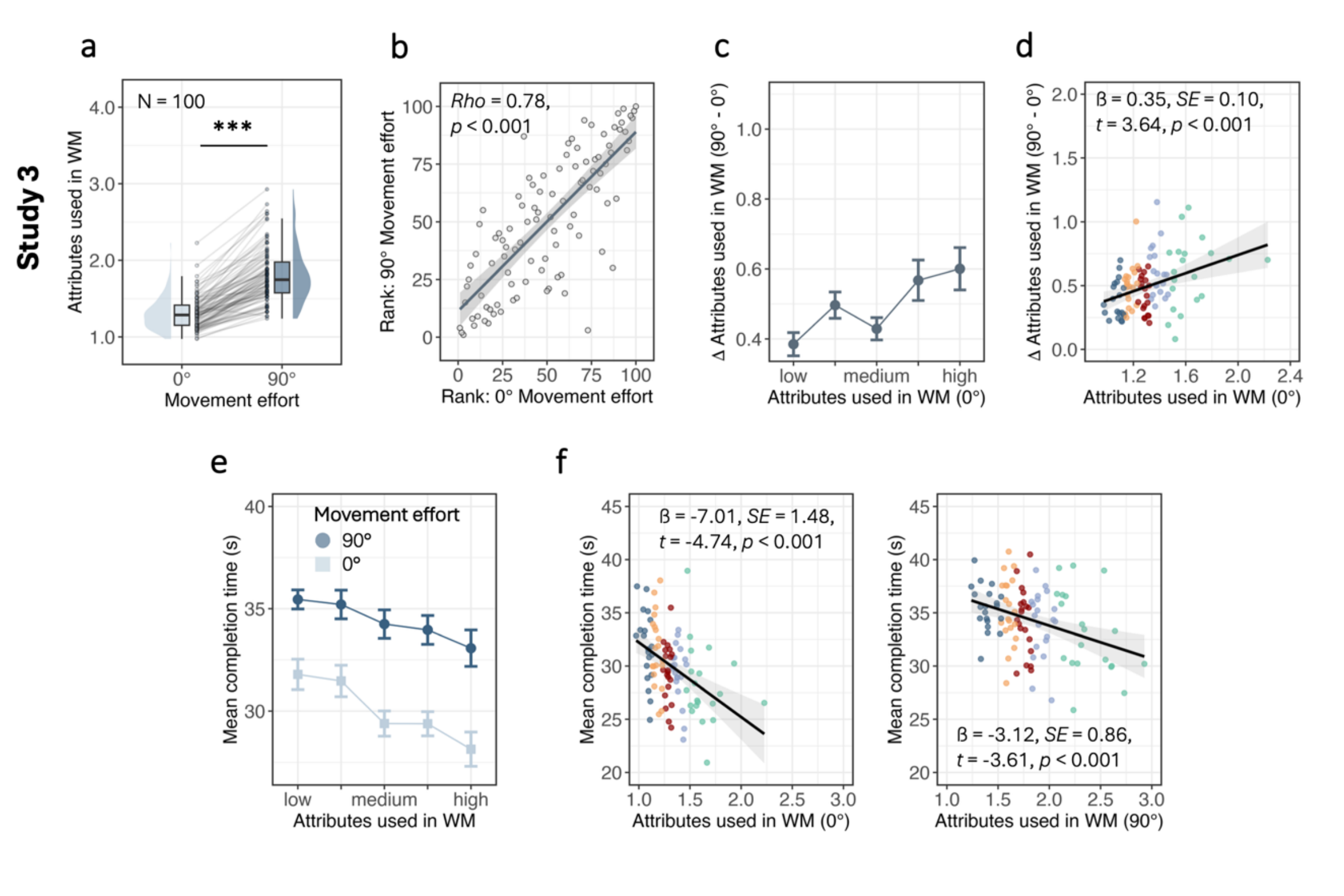

***Figure S4*. Participant specific tendencies in WM-usage, replication with data from Study 3 only. a)** Intra-individual variability in WM-usage: Consistent with the combined analysis, participants in Study 2 used more attributes in WM when movement effort was high compared to low (t = -22.84, df = 99, p < 0.001; d = -2.28, CI95% = [-2.66, -1.91]). Dots and lines represent individual participant data. **b)** Systematic differences in participants spontaneous WM-usage: Consistent with the combined analysis, participants' relative WM-usage (ranked average attributes used in WM) was highly correlated across movement effort conditions (*Rho* = 0.78, p < 0.001) in Study 1. Dots represent individual participant data. **c) and d)** Consistent with the combined analysis, we found a positive relation between participants' WM-usage in low-movement effort trials and their increase in WM-usage when movement effort increased (β = 0.35, SE = 0.10, t =3.64, p < 0.001). Panel c visualises participants' categorical WM-usage in low-movement effort trials. Here, error bars depict standard errors (N = 20 per group). Panel d depicts participants' WM-usage in low-movement effort trials without categorical grouping. Here, dots represent individual participant data. Colour indicates matching to categorical groups. **e) and f)** Consistent with the combined analysis, we found a negative relationship between participants average attributes used in WM and their task efficiency as indexed by average trial completion time when movement effort was low (β = -7.01, SE = 1.48, t =-4.74, p < 0.001) and high (β = -3.12, SE = 0.86, t =-3.61, p < 0.001). Panel e visualises participants' WM-usage as categorical means (N = 20 per group). Here, error bars depict standard errors. Panel f depicts participants' WM-usage without categorical grouping. Here, dots represent individual participant data. Colour indicates matching to categorical groups.

Table S2. Descriptive statistics Study 3.

Task/Measure	N	Mean	SD	Skew	Kurtosis	Reliability
Attributes used in WM (90)	100	1.81	0.35	0.88	3.61	0.96
Attributes used in WM (0)	100	1.31	0.21	1.27	5.70	0.95
Operation span	99	66.60	7.72	-1.20	4.05	0.56
Rotation span	99	31.13	6.31	-0.71	3.13	0.70
Symmetry span	99	34.23	5.71	-0.80	2.99	0.59
Stroop Squared	95	37.03	13.65	-0.67	3.14	0.94
Simon Squared	100	64.59	9.97	-0.55	3.47	0.93
Flanker Squared	100	36.07	12.38	1.19	3.99	0.95

Note. Reliability estimates represent mean of *N* = 1000 random split-half reliability corrected using Spearman-Brown prophecy formula.

Table S3. Task level correlations (Study 3)

Task/Measure	1	2	3	4	5	6	7	8	9	10
1. Attributes used in WM (90)	—									
2. Attributes used in WM (0)	**.82**	—								
3. Operation span	.12	-.01	—							
4. Rotation span	**.31**	**.26**	**.43**	—						
5. Symmetry span	**.20**	**.20**	**.56**	**.60**	—					
6. WMC composite	**.25**	.18	**.81**	**.82**	**.87**	—				
7. Stroop Squared	**.26**	**.28**	**.26**	**.39**	**.39**	**.40**	—			
8. Simon Squared	.17	**.26**	**.41**	**.52**	**.52**	**.58**	**.52**	—		
9. Flanker Squared	**.21**	**.29**	**.41**	**.54**	**.43**	**.55**	**.58**	**.59**	—	
10. AC composite	**.26**	**.34**	**.43**	**.58**	**.53**	**.61**	**.83**	**.84**	**.86**	—

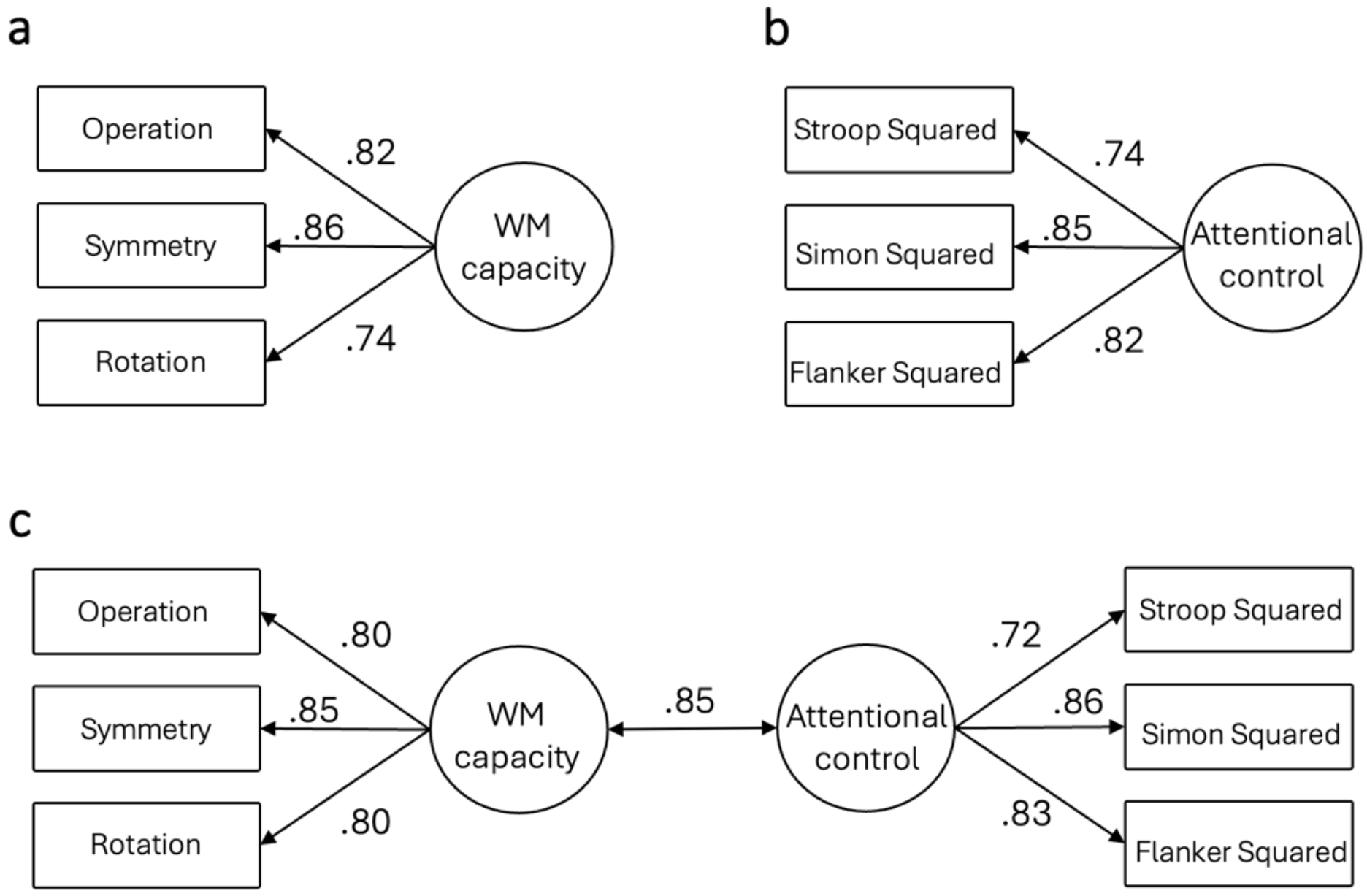

Figure S5. Confirmatory factor analyses for AC and WMC tasks. a) CFA testing whether all three WM capacity task load onto a common factor ($\chi2(1) = 8.38$, $p = .004$; $CFI = 0.91$, $TLI = 0.72$, $RMSEA = 0.27$ ($CI_{90\%} = [0.13, 0.45]$), $SRMR = 0.25$). All three tasks loaded strongly onto a common factor, but fit indices suggested an overall poor model fit. **b)** CFA testing whether all three attentional control tasks load onto a common factor ($\chi2(1) = 6.73$, $p = .009$; $CFI = 0.93$, $TLI = 0.80$, $RMSEA = 0.24$ ($CI_{90\%} = [0.09, 0.42]$), $SRMR = 0.21$). All three tasks loaded strongly onto a common factor, but fit indices suggested an overall poor model fit. **c)** Two-factor model with a latent factor for WM capacity and attentional control ($\chi2(10) = 22.60$, $p = .01$; $CFI = 0.94$, $TLI = 0.91$, $RMSEA = 0.11$ ($CI_{90\%} = [0.05, 0.17]$), $SRMR = 0.28$), showing mixed model fit. All tasks again loaded strongly on the latent factors. The latent factors for attentional control and WM capacity covaried ($\beta = 0.85$, $SE = 0.05$, $z = 16.33$, $p < .001$), indicating a strong relationship between the two variables. Given that the models demonstrated mixed or poor fit (possibly reflecting sample size limitations for this approach and/or model misspecifications), we interpret these results with caution.

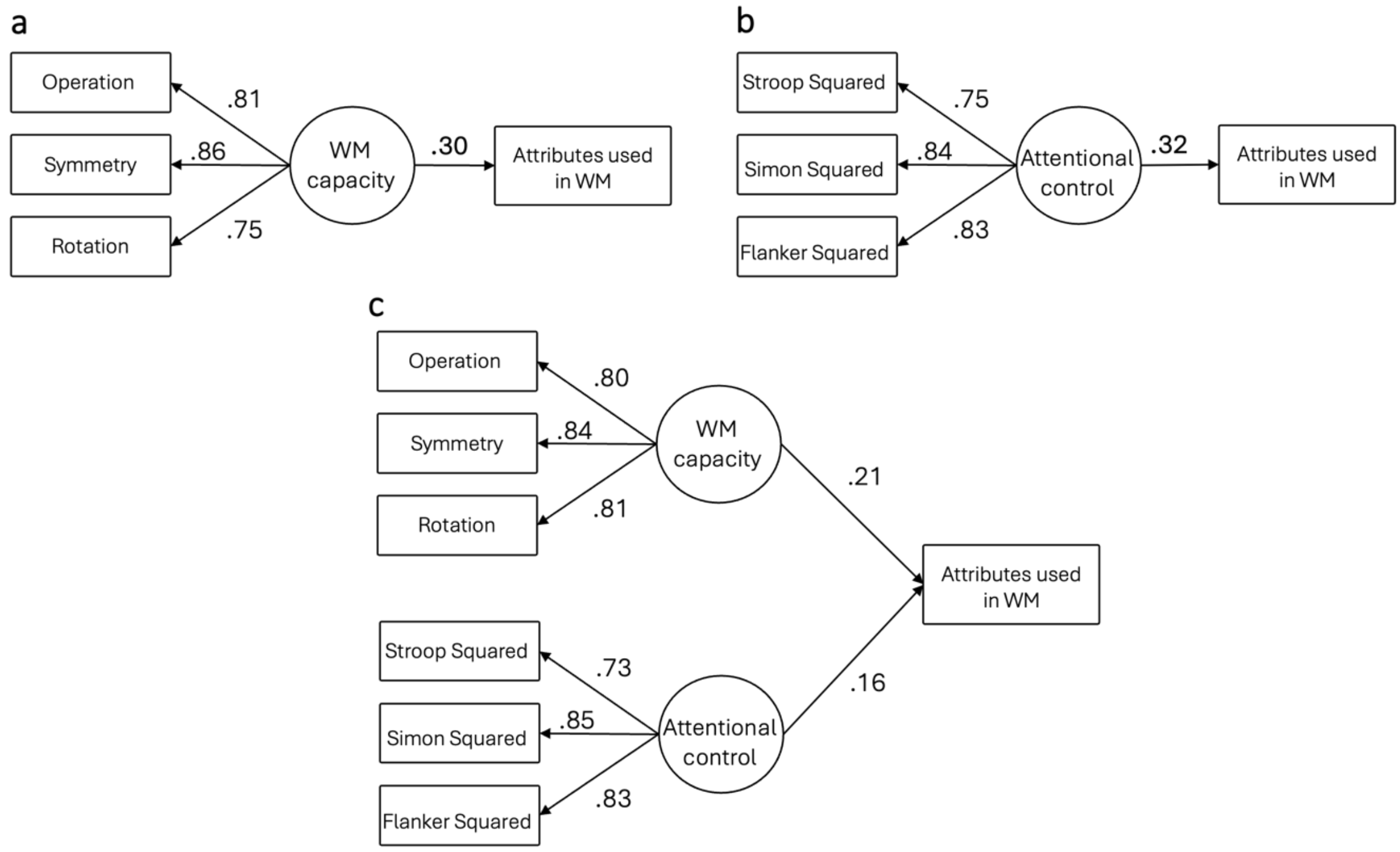

Figure S6. Predicting WM usage by attentional control and WM capacity in a SEM framework. a) SEM with a latent factor for WM capacity predicting participants' average attributes used in WM ($\chi2(3) = 12.43$, p = .006; *CFI* = 0.89, *TLI* = 0.78, *RMSEA* = 0.17 ($CI_{90\%}$ = [0.08, 0.28]), *SRMR* = 0.21), showing poor model fit. A significant positive relationship was found between WM capacity and participants' average attributes used in WM (ß = 0.30, *SE* = 0.12, *z* = 2.46, *p* = .01). **b)** SEM with a latent factor for AC predicting participants' average attributes used in WM ($\chi2(3) = 8.50$, p = .04; *CFI* = 0.94, *TLI* = 0.88, *RMSEA* = 0.14 ($CI_{90\%}$ = [0.03, 0.25]), *SRMR* = 0.18), showing mixed model fit. A significant positive relationship was found between AC and participants' average attributes used in WM (ß = 0.32, *SE* = 0.12, *z* = 2.56, *p* = .01). **c)** SEM with latent factors for both WM capacity and attentional control predicting participants' average attributes used in WM ($\chi2(14) = 27.93$, p = .01; *CFI* = 0.94, *TLI* = 0.91, *RMSEA* = 0.10 ($CI_{90\%}$ = [0.04, 0.15]), *SRMR* = 0.26), showing mixed model fit. No significant relationship was found between AC (ß = 0.16, *SE* = 0.27, *z* = 0.59, *p* = .55) or WM capacity (ß = 0.21, *SE* = 0.27, *z* = 0.78, *p* = .44) and participants' average attributes used in WM. Given that the models demonstrated mixed or poor fit (possibly reflecting sample size limitations for this approach and/or model misspecifications), we interpret these results with caution.

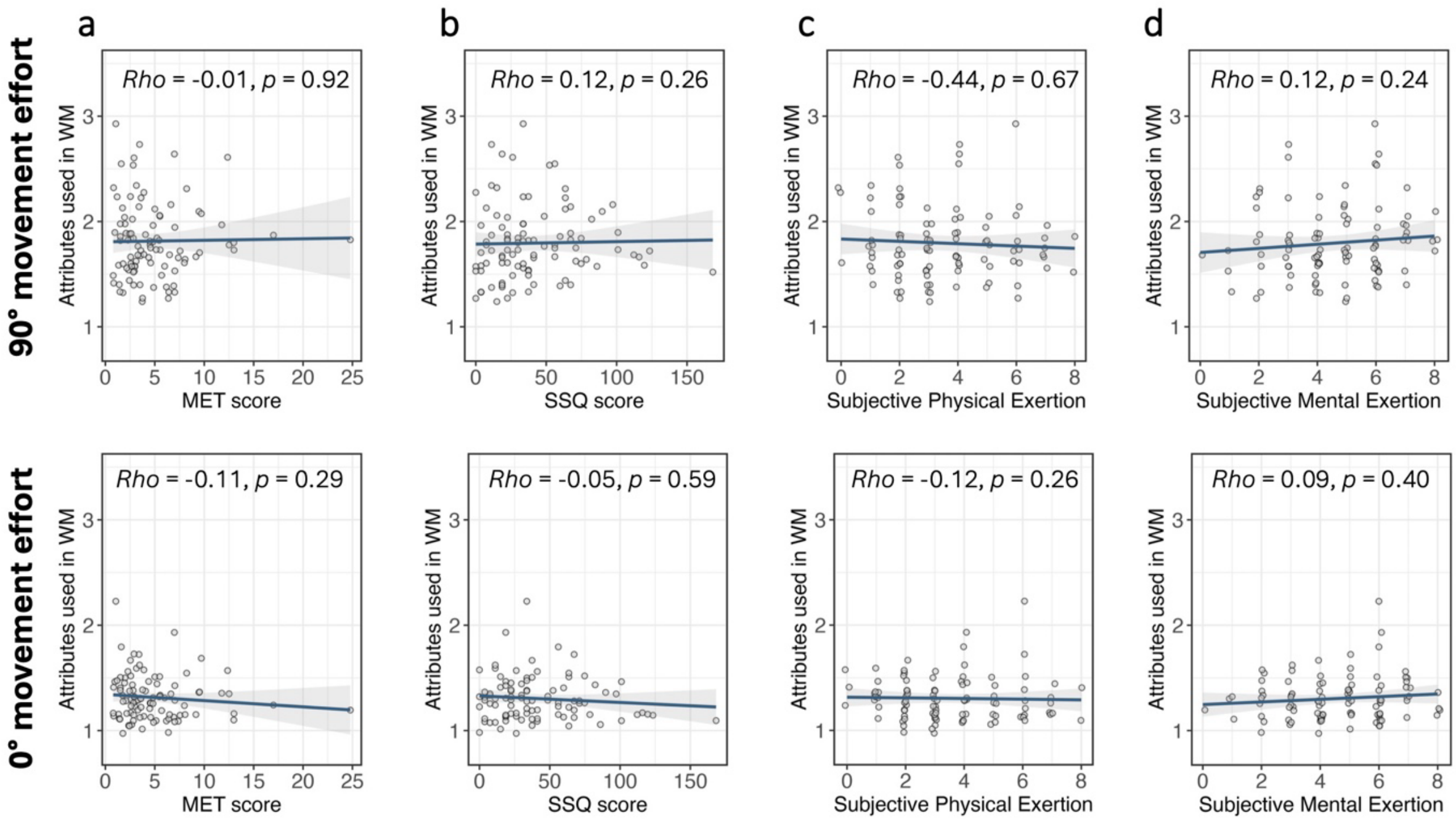

Figure S7. **Exploratory analysis in Study 3. a)** We found no meaningful relationship between MET scores (cite IPAQ) and participants' average attributes used in memory, neither when movement effort was high (*Rho* = -.01, *p* = 0.92) or low (*Rho* = -.12, *p* = 0.25). **b)** We found no meaningful relationship between SSQ scores and participants' average attributes used in memory, neither when movement effort was high (*Rho* = .10, *p* = 0.32) or low (*Rho* = -.05, *p* = 0.32). **c)** We found no meaningful relationship between subjective physical exertion during the VR task and participants' average attributes used in memory, neither when movement effort was high (*Rho* = .05, *p* = 0.64) or low (*Rho* = -.11, *p* = 0.30). **d)** We found no meaningful relationship between subjective mental exertion during the VR task and participants' average attributes used in memory, neither when movement effort was high (*Rho* = .11, *p* = 0.28) or low (*Rho* = .10, *p* = 0.35).
